# Heterozygous mutations in the C-terminal domain of COPA underlie a complex autoinﬂammatory syndrome

**DOI:** 10.1172/JCI163604

**Published:** 2024-01-04

**Authors:** Selket Delafontaine, Alberto Iannuzzo, Tarin M. Bigley, Bram Mylemans, Ruchit Rana, Pieter Baatsen, Maria Cecilia Poli, Daisy Rymen, Katrien Jansen, Djalila Mekahli, Ingele Casteels, Catherine Cassiman, Philippe Demaerel, Alice Lepelley, Marie-Louise Frémond, Rik Schrijvers, Xavier Bossuyt, Katlijn Vints, Wim Huybrechts, Rachida Tacine, Karen Willekens, Anniek Corveleyn, Bram Boeckx, Marco Baggio, Lisa Ehlers, Sebastian Munck, Diether Lambrechts, Arnout Voet, Leen Moens, Giorgia Bucciol, Megan A. Cooper, Carla M. Davis, Jérôme Delon, Isabelle Meyts

**Affiliations:** 1 Laboratory for Inborn Errors of Immunity, Department of Microbiology, Immunology and Transplantation, KU Leuven, Leuven, Belgium.; 2 Department of Pediatrics, University Hospitals Leuven, Leuven, Belgium.; 3 Université Paris Cité, CNRS, INSERM, Institut Cochin, Paris, France.; 4 Department of Pediatrics, Division of Rheumatology/Immunology, Washington University in St. Louis, St. Louis, Missouri, USA.; 5 Laboratory of Biomolecular Modelling and Design, Department of Chemistry, KU Leuven, Leuven, Belgium.; 6 Division of Immunology, Allergy and Retrovirology, Baylor College of Medicine, Texas Children’s Hospital, Houston, Texas, USA.; 7 Electron Microscopy Platform of VIB Bio Imaging Core, KU Leuven, Leuven, Belgium.; 8 Department of Pediatrics, Clínica Alemana de Santiago, Universidad del Desarollo, Santiago, Chile.; 9 Immunology and Rheumatology Unit, Hospital de Niños Dr. Roberto del Rio, Santiago, Chile.; 10 PKD Research Group, Laboratory of Ion Channel Research, Department of Cellular and Molecular Medicine, KU Leuven, Leuven, Belgium.; 11 Department of Pediatric Nephrology,; 12 Department of Ophthalmology, and; 13 Department of Radiology, University Hospitals Leuven, Leuven, Belgium.; 14 Université Paris Cité, Imagine Institute, Laboratory of Neurogenetics and Neuroinﬂammation, INSERM UMR 1163, Paris, France.; 15 Paediatric Haematology-Immunology and Rheumatology Unit, Necker Hospital, AP-HP.Centre - Université Paris Cité, Paris, France.; 16 Allergy and Clinical Immunology Research Group, Department of Microbiology, Immunology and Transplantation, and; 17 Clinical and Diagnostic Immunology, Department of Microbiology, Immunology and Transplantation, KU Leuven, Leuven, Belgium.; 18 Center for Human Genetics, Leuven University Hospitals, Leuven, Belgium.; 19 Laboratory for Translational Genetics, Department of Human Genetics, KU Leuven, Leuven, Belgium.; 20 VIB Center for Cancer Biology, Leuven, Belgium.; 21 VIB Bio Imaging Core and VIB–KU Leuven Center for Brain & Disease Research, KU Leuven Department of Neurosciences, Leuven, Belgium.

**Keywords:** Immunology, Cell stress, Innate immunity, Monogenic diseases

## Abstract

Mutations in the N-terminal WD40 domain of coatomer protein complex subunit α (COPA) cause a type I interferonopathy, typically characterized by alveolar hemorrhage, arthritis, and nephritis. We described 3 heterozygous mutations in the C-terminal domain (CTD) of COPA (p.C1013S, p.R1058C, and p.R1142X) in 6 children from 3 unrelated families with a similar syndrome of autoinflammation and autoimmunity. We showed that these CTD COPA mutations disrupt the integrity and the function of coat protein complex I (COPI). In COPA^R1142X^ and COPA^R1058C^ fibroblasts, we demonstrated that COPI dysfunction causes both an anterograde ER-to-Golgi and a retrograde Golgi-to-ER trafficking defect. The disturbed intracellular trafficking resulted in a cGAS/STING-dependent upregulation of the type I IFN signaling in patients and patient-derived cell lines, albeit through a distinct molecular mechanism in comparison with mutations in the WD40 domain of COPA. We showed that CTD COPA mutations induce an activation of ER stress and NF-κB signaling in patient-derived primary cell lines. These results demonstrate the importance of the integrity of the CTD of COPA for COPI function and homeostatic intracellular trafficking, essential to ER homeostasis. CTD COPA mutations result in disease by increased ER stress, disturbed intracellular transport, and increased proinﬂammatory signaling.

## Abstract 2

<p>Mutations in the C-terminal domain of COPA impair intracellular transport through COPI dysfunction and result in a concerted activation of several pro-inflammatory signaling pathways.</p>

## Graphical abstract

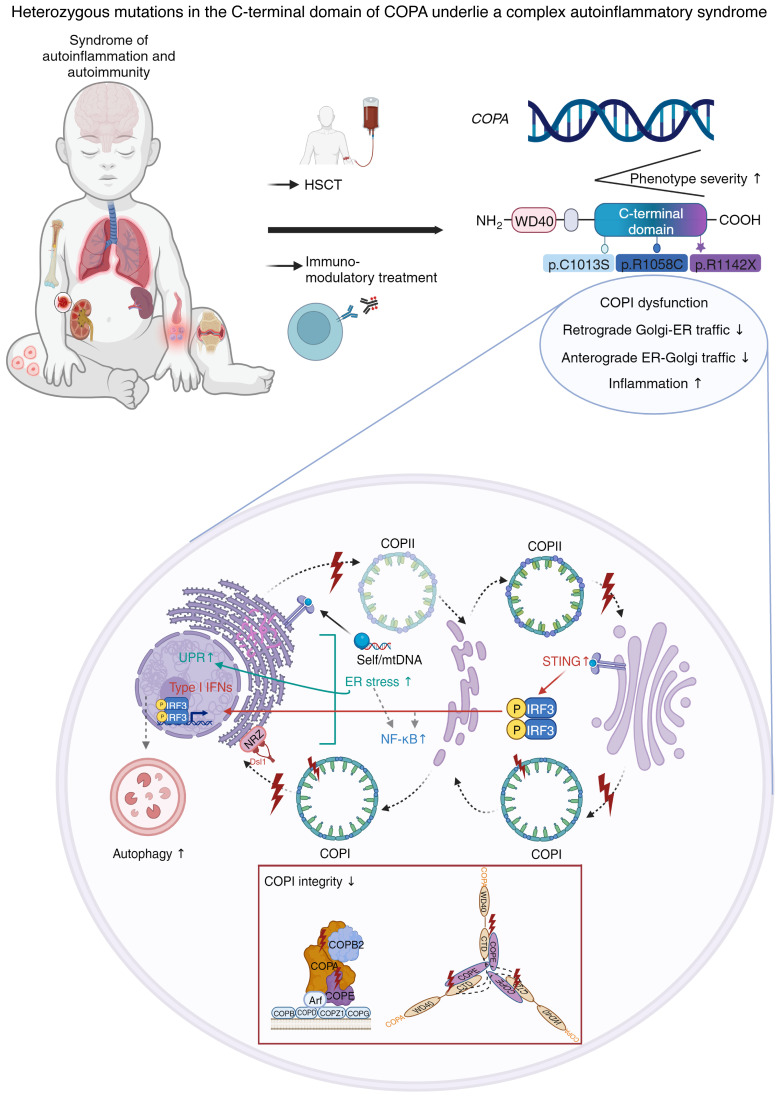

## Introduction

The COPA gene encodes coatomer protein complex subunit α (COPA) (1). COPA was described in 1991 as a coat subunit of Golgi-derived non-clathrin-coated vesicles, later termed “coatomer” (2, 3). Coatomer or coat protein complex I (COPI) is ubiquitously expressed and localizes to the Golgi apparatus, cytosol, and endoplasmic reticulum (ER) (4, 5). In mammalian cells, subunits α, β′, and ε form the outer-coat subcomplex, while the adaptor subcomplex comprises the β, γ, δ, and ζ subunits (6). Upon activation by brefeldin A–sensitive guanine nucleotide exchange factor (GEF), coatomer is recruited to the target membrane, where it captures cargo molecules and polymerizes into spherical cages that mold the membrane into a COPI-coated bud (7, 8). Ultimately, cargo-incorporated COPI vesicles are released in the cytosol (9). COPI and coat protein complex II (COPII) are essential for the intracellular vesicular transport of proteins in eukaryotic cells (10). While COPII transports newly synthesized proteins from the ER to the Golgi apparatus, COPI retrieves ER-resident cargo proteins from the Golgi apparatus or ER-Golgi intermediate compartment (ERGIC) to the ER to ensure their steady-state distribution (11, 12). Recently, COPI has been discovered to be required for early endosome maturation, autophagy, and lysosomal trafficking (13, 14).

In 2015, heterozygous dominant-negative mutations affecting a narrow stretch of 14 amino acids in the WD40 domain located in the N-terminal domain of COPA were shown to underlie autosomal dominant COPA syndrome (Online Mendelian Inheritance in Man [OMIM] 616414) (15). Patients typically present with a triad of interstitial lung disease with or without pulmonary hemorrhage, inﬂammatory arthritis, and immune complex–mediated nephropathy (16). This condition displays incomplete penetrance. Seventy-five patients with mutations in the N-terminal domain of COPA have been described until now, with great variability in terms of both age of onset and clinical severity (15, 16, 17–33). The mutations do not affect COPA protein expression, but they impair the retrograde Golgi-to-ER retrieval of dilysine-tagged ER-resident proteins, such as surfeit locus protein 4 (SURF4) and, indirectly, stimulator of interferon genes (STING) (17, 34, 35). STING is a crucial immune sensor for multiple cytoplasmic nucleic acid sensors such as cyclic GMP-AMP synthase (cGAS) (36). Upon sensing of double-stranded DNA, cGAS synthesizes 2′-3′-cyclic GMP-AMP (2′3′-cGAMP), which binds STING and induces STING dimerization and its ER-to-Golgi translocation in COPII vesicles (37). Downstream signaling through TANK-binding kinase 1–mediated (TBK1-mediated) activation of transcription factors NF-κB and IFN regulatory factor 3 (IRF3) results in the expression of proinﬂammatory cytokines including type I and III interferon (IFN) (36). Failure of STING retrieval to the ER causes its constitutive activation at the Golgi and constitutive type I IFN signaling (17). In overexpression systems, this type I IFN signaling was demonstrated to have a variable cGAS dependency (17, 36). Furthermore, expression of mutant COPA results in ER stress, impaired autophagy, and a skewing of CD4^+^ T cells toward a T helper type 17 (Th17) phenotype (15).

The C-terminal domain (CTD) of COPA remains less well defined. It is essential for COPI integrity through the binding of coatomer protein complex subunit ε (COPE) and the homo-oligomerization of COPA-COPE dimers (38). Additionally, the CTD plays a role in vesicle tethering to the ER (39, 40). In yeast cells, dysfunction and instability of COPI were induced by, respectively, a deletion of 170 residues and a point mutation in the CTD of COPA (38, 41). Here we investigate and functionally validate 3 heterozygous mutations in the CTD of COPA in 6 patients from 3 unrelated families.

## Results

### Autoinﬂammation and autoimmunity in 6 children from 3 unrelated families.

**Figure 1:**
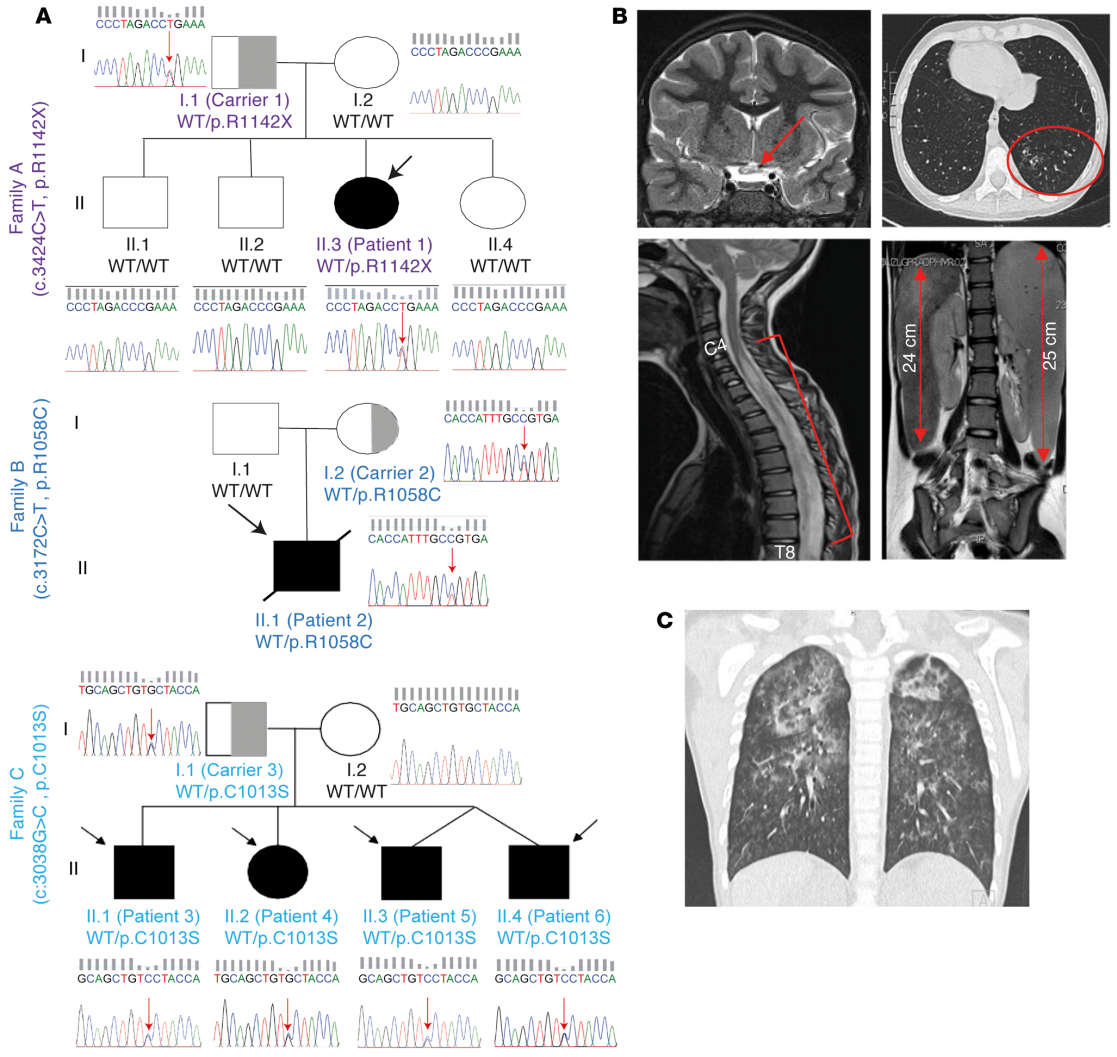
Three heterozygous mutations in the CTD of COPA in 6 patients with an autoinﬂammatory and autoimmune phenotype. (**A**) Pedigrees of families A, B, and C with indication of the genotype, assigned with the amino acid changes, below each individual. Affected individuals are indicated by black filled symbols and an arrow, gray half-filled symbols indicate unaffected heterozygous carriers, and open shapes indicate unaffected family members. Squares designate males and circles females. Patient 2 (B.II.1) died at the age of 7 due to hemophagocytic lymphohistiocytosis and multiple organ failure. Patients 5 (C.II.3) and 6 (C.II.4) are dizygotic twins. Sanger sequencing chromatograms for COPA, performed on genomic DNA, are shown, covering a 15 bp snapshot around the mutation. Red arrows indicate the position of the mutation. (**B**) Medical imaging for patient 1 (A.II.3). Brain MRI (T2-weighted images) shows a hyperintense optic chiasm (red arrow), indicative of neuromyelitis optica with involvement of the optic chiasm (top left panel). Spinal cord MRI (T2 weighted images) illustrates a swollen spinal cord with central hyperintensity (red bracket), ranging from the level of cervical vertebra 4 (C4) to thoracic vertebra 8 (T8), revealing transverse myelitis (bottom left panel). Chest computed tomography (CT) demonstrates bronchiectasis (top right panel), and MRI of the abdomen depicts hepatosplenomegaly (bottom right panel, red arrows). (**C**) Chest CT of patient 2 (B.II.1) shows signs of an alveolar hemorrhage and centrilobular nodules.

We identified 6 patients in 3 unrelated families (Figure 1; Supplemental Tables 1 and 5; Supplemental Figure 1; and full case reports in Supplemental Methods; supplemental material available online with this article; https://doi.org/10.1172/JCI163604DS1). The phenotype of these patients ranged from extremely severe (2/6 patients) to mild (4/6 patients). Patient 1 (A.II.3) presented at the age of 8 years with anti–aquaporin-4 antibody–positive neuromyelitis optica (Figure 1B). During the following years, she experienced relapses of neuromyelitis optica and transverse myelitis (Figure 1B). Despite extensive immunosuppressive regimens, she developed hepatosplenomegaly, cirrhosis, intra-abdominal lymphadenopathy, progressive pancytopenia, and alveolar hemorrhage (Figure 1B). At the age of 15 years 6 months, she received a hematopoietic stem cell transplantation. Patient 2 (B.II.1) presented at the age of 5 years with livedo reticularis, acrocyanosis, heat hypersensitivity, somnolence, and an unusual sleep-wake cycle. He experienced recurrent alveolar hemorrhage, unresponsive to extensive immunomodulatory treatment (Figure 1C). He died 2 years after his initial presentation due to secondary hemophagocytic lymphohistiocytosis and multiple organ failure. Notably, none of the patients with WD40 COPA mutations have died outside the context of lung transplantation. Patient 3 (C.II.1) presented at 5 years old with IgA nephropathy, which necessitated prolonged immunosuppressive treatment. His sister, patient 4 (C.II.2), was affected by a neuroblastoma at age 12 years. During follow-up she developed arterial hypertension, stage II kidney disease, complex regional pain syndrome, and vasculitis of the legs. Patients 5 (C.II.3) and 6 (C.II.4), prematurely born dizygotic twins, both had recurrent upper respiratory tract infections. Patient 6 (C.II.4) developed a neonatal alveolar hemorrhage. All siblings of family C had recurrent arthralgia, which resolved with nonsteroidal antiinﬂammatory drugs. Autoantibodies were detected in 3 of 6 patients (Supplemental Table 2). Patient 1 (A.II.3) showed a high percentage of HLA-DR–positive T cells and a low percentage of naive T and B cells, prior to treatment with rituximab (Supplemental Table 3). Furthermore, CD4^+^ T cells of patient 1 (A.II.3) did not demonstrate a significant increase in the frequency of Th17 cells (Supplemental Figure 2A). Immunophenotyping of patient 2 (B.II.1) was unremarkable. Patient 3 (C.II.1) had a mildly elevated percentage of HLA-DR–positive T cells, and patient 4 (C.II.2) a low percentage of B cells. Naive T cell percentages were low for patients 3–5 (C.II.1–3). Immunophenotyping of patient 6 (C.II.4) was unavailable (Supplemental Table 3) (42).

### Three heterozygous mutations in the CTD of COPA in patients.

**Figure 2:**
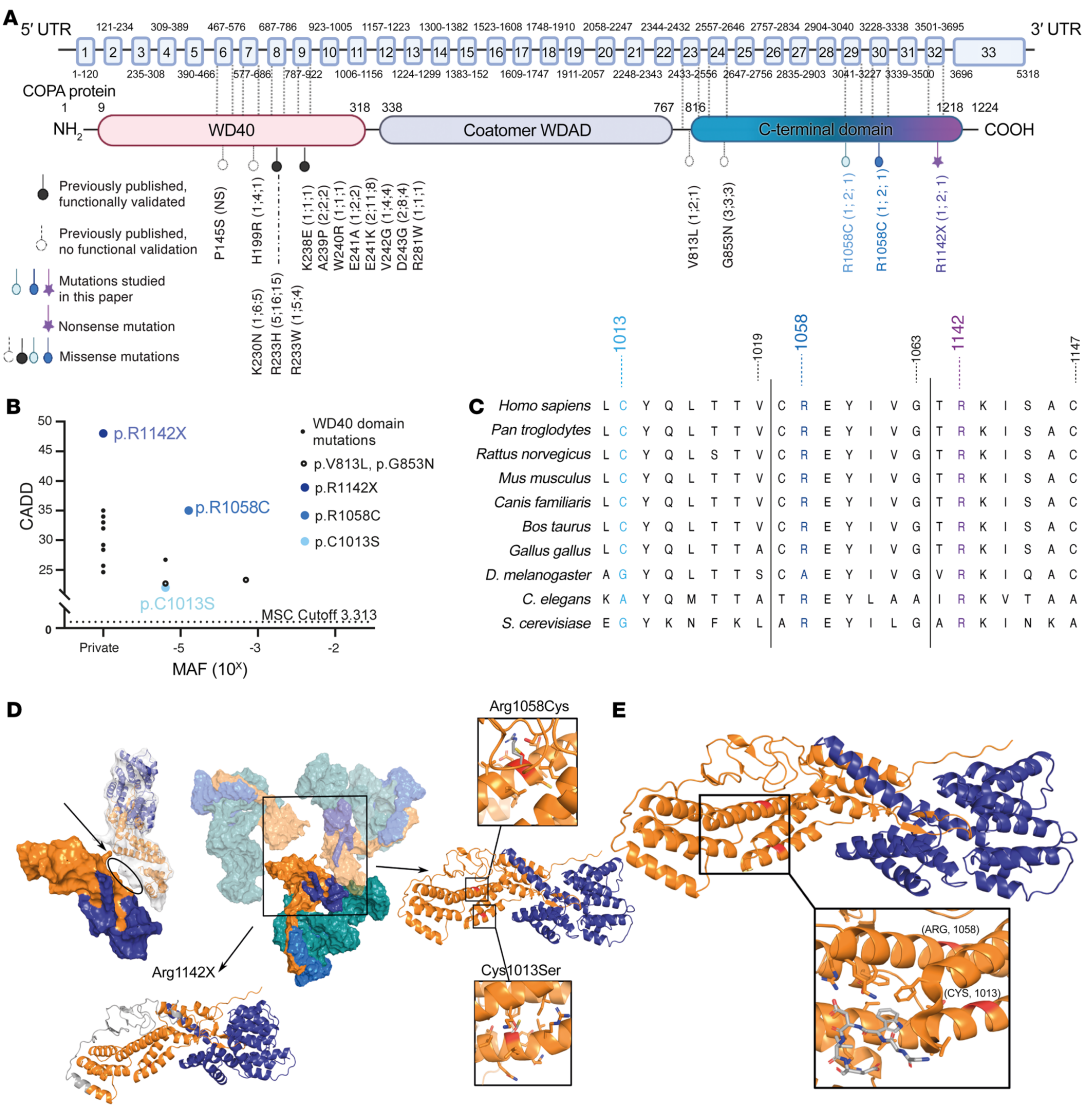
Genetic aspects and in silico pathogenicity prediction of mutations in the CTD of COPA. (**A**) Schematic illustration of the COPA protein and its domains. Previously reported mutations in the WD40 domain are depicted in black (filled circle, functionally validated; open circle, functional validation unavailable), mutations in the CTD of COPA in color (color code correlates with Figure 1A). Numbers in parentheses refer to the number of families identified, number of mutation carriers, and number of diseased, respectively. Coatomer WDAD, coatomer WD-associated region. (**B**) Population genetics of the previously described COPA mutations affecting the WD40 domain, previously published not functionally validated CTD mutations, and CTD COPA mutations. MAF, minor allele frequency; MSC, mutation significance cutoff; CADD, combined annotation-dependent depletion score. (**C**) Conserved sequence homology at the site of the identified mutations in distantly related eukaryotes. (**D**) Biomodeling of the mutations affecting the CTD of COPA. The central figure depicts the main proteins of COPI, COPA (orange), COPB (teal), COPB2 (blue), and COPE (purple). Left: The physical interaction between COPA^R1142X^ and COPA^WT^ is depicted. In the top representation, COPA^WT^ is shown as surface and COPA^R1142X^ as a cartoon inside the surface, illustrating complete removal of the dimerization interface of COPA^R1142X^ with the neighboring COPA^WT^ (oval). This exposes the hydrophobic interface of the COPE binding helices, thus disrupting the COPA-COPE dimer. In the bottom representation, the absent residues are colored in gray. Right: The interaction between COPA^R1058C^, COPA^C1013S^, and COPE is shown. COPA^R1058C^ and COPA^C1013S^ likely disturb the conformation of the α-helices on which they are located and subsequently disrupt COPA’s overall structure. (**E**) Magnification of biomodeling of the α-helices, which compose the main body of the CTD and comprise residues 1013 and 1058, and form a binding site for singleton tryptophan motif (STM). STMs are known to be crucial for COPA homo-oligomerization and ER tethering of COPI vesicles.

Whole-exome sequencing was performed on all patients and their parents, except patient 6 (C.II.4) and B.I.1. Three heterozygous mutations, 1 nonsense and 2 missense, exclusively affecting the CTD of COPA, were detected (Figure 2A). Patient 1 (A.II.3) and her father (carrier 1, A.I.1) were heterozygous for the c.3424C>T (p.R1142X) variant. This variant was private and was predicted to be deleterious through the generation of a premature stop codon. Patient 2 (B.II.1) and his mother (carrier 2, B.I.2) were heterozygous for the c.3172C>T (p.R1058C) variant. The 4 patients of family C (patients 3–6, C.II.1–4) and their father (carrier 3, C.I.1) were heterozygous for the c.3038G>C (p.C1013S) variant. The missense mutations, p.R1058C and p.C1013S, had a respective allele frequency of 0.000017 and 0.000010 in the Genome Aggregation Database (gnomAD) and were predicted to be pathogenic by in silico prediction algorithms, which attributed a greater pathogenic potential to the p.R1058C mutation compared with the p.C1013S mutation (Figure 2B and Supplemental Table 4) (43–46). Sequence homology was highly conserved at the site of the identified mutations among distantly related eukaryotes (Figure 2C). All variants were confirmed by Sanger sequencing (Figure 1A).

**Figure 3:**
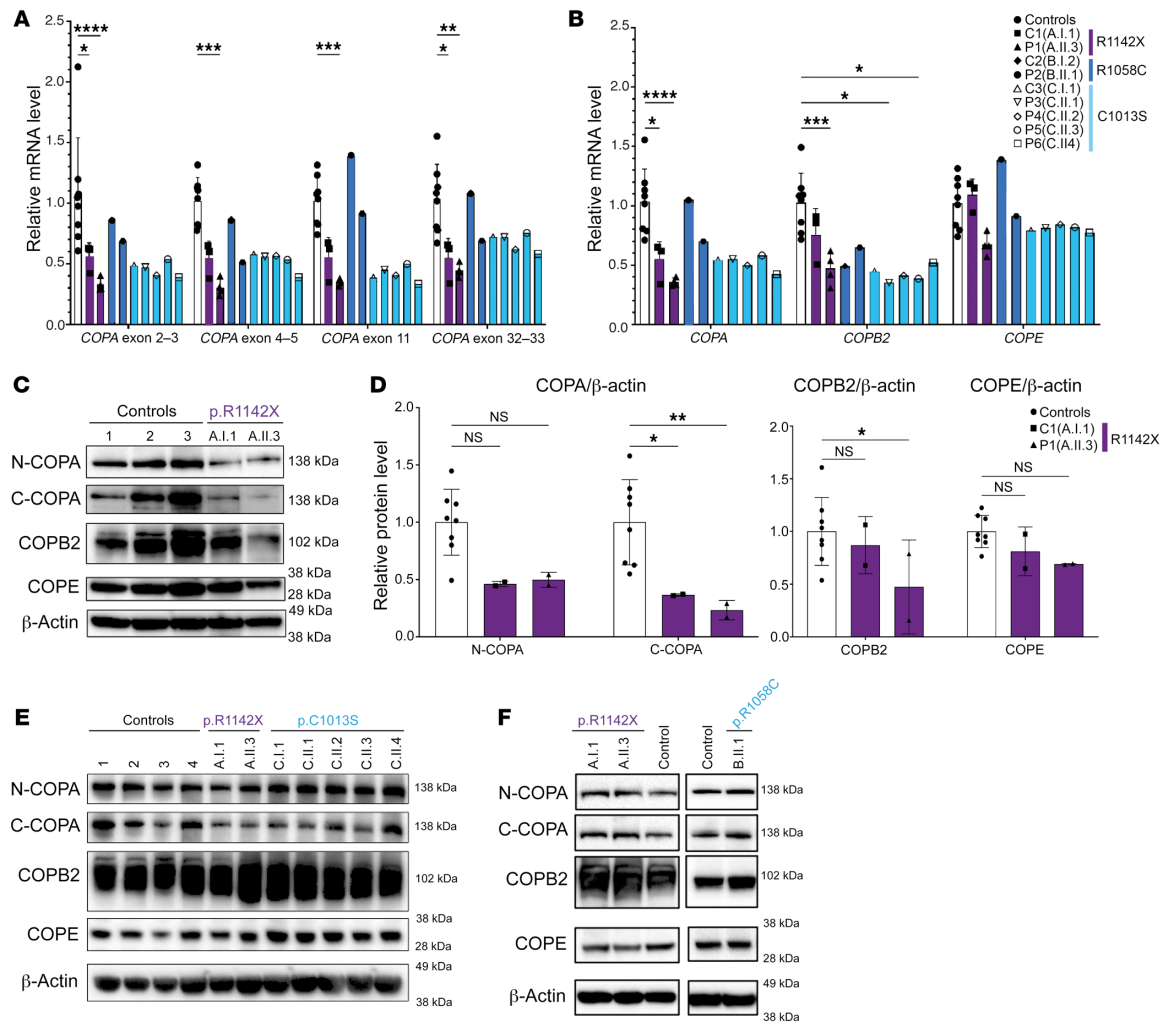
Analysis of COPA, COPB2, and COPE mRNA and protein expression in patients affected by mutations in the CTD of COPA. (**A** and **B**) RT-qPCR analysis of transcript levels of COPA, COPB2, and COPE analyzed in cDNA extracted from whole blood (for patients and carriers of families A and C) or PBMCs (for B.I.2 and B.II.1). (**A**) COPA mRNA expression, evaluated using 4 probes covering exons 2–3, 4–5, 11, and 32–33. (**B**) Transcript levels of COPA (calculated as mean of transcript levels of the 4 COPA probes, shown in **A**), COPB2, and COPE. Relative mRNA level depicts fold increase of gene expression normalized to GAPDH (ΔCt) and to mean ΔCt of control samples. Three to four samples from separate time points were analyzed for A.II.3 and A.I.1. (**C**) Western blot analysis of COPA, COPB2, and COPE in whole-cell lysates of PBMCs of A.I.1, A.II.3, and 3 healthy controls. Immunoblotting of COPA with an antibody specific for the N-terminal region of COPA (N-COPA) and an antibody specific for the C-terminal region of COPA (C-COPA). (**D**) Quantification of protein level of COPA, detected by N-COPA and C-COPA antibody (left), and COPB2 and COPE (right), as observed in **C**. Band intensity was determined relative to GAPDH and normalized to mean of healthy controls (*n* = 8, 5 adults, 3 children). (**E**) Western blot analysis of COPA, COPB2, and COPE in EBV LCLs of A.I.1, A.II.3, C.I.1, and C.II.1–4 compared with 4 adult healthy controls. (**F**) Western blot analysis of COPA, COPB2, and COPE in fibroblasts of A.I.1, A.II.3, and B.II.1 compared with 2 healthy controls. Results in **C**–**F** are representative of 2–3 independent experiments. In **A**, **B**, and **D**, columns and bars represent mean ± SEM values, statistically analyzed using 2-way ANOVA (*P < 0.05; **P < 0.01; ***P < 0.001; ****P < 0.0001).

First, we analyzed the impact of these mutations on the expression of COPA, and its closest binding partners, COPB2 and COPE, at both the mRNA and the protein level. Quantitative reverse transcription PCR (RT-qPCR), using 4 probes covering exons 2–3, exons 4–5, exon 11, and exons 32–33 of COPA, revealed a significantly reduced COPA level, in cDNA extracted from whole blood of patient 1 (A.II.3), for each COPA probe (Figure 3A). Subsequently, the mean COPA level, expressed as the mean of the COPA levels determined using the 4 COPA probes, demonstrated a significantly reduced COPA mRNA level in patient 1 (A.II.3) and carrier 1 (A.I.1) (Figure 3B). Based on these results, we suspected nonsense-mediated decay of the COPA^R1442X^ allele, although Sanger sequencing of COPA cDNA extracted from whole blood of patient 1 (A.II.3) and carrier 1 (A.I.1) detected wild-type (WT) and mutant COPA transcripts (Supplemental Figure 2B). Furthermore, COPA mRNA levels tended to be reduced in patient 2 (B.II.1), patients 3–6 (C.II.1–4), and carrier 3 (C.I.1), while carrier 2 (B.I.2) demonstrated levels in the range of controls (Figure 3A). COPB2 mRNA expression was significantly reduced in whole blood of patient 1 (A.II.3), patient 3 (C.II.1), and patient 5 (C.II.3), and tended to be reduced for the remaining patients and carriers (Figure 3B). The COPE expression tended to be reduced for all patients and carrier 3 (C.I.1) (Figure 3B). In contrast, COPA, COPB2, and COPE mRNA levels did not significantly differ from controls in patients’ and carriers’ EBV-transformed B lymphoblastoid cell lines (EBV LCLs) and fibroblasts (Supplemental Figure 3, A and B).

Next, protein expression of COPA was analyzed in peripheral blood mononuclear cells (PBMCs) of patient 1 (A.II.3) and carrier 1 (A.I.1). COPA expression in COPA^R1142X^ PBMCs, analyzed with an antibody specific for the N-terminal region of COPA, tended to be reduced, while immunoblotting with an antibody targeted against the C-terminal amino acid 1150 of COPA revealed a significantly reduced COPA expression. Further, COPB2 protein level was reduced in PBMCs of patient 1 (A.II.3), and finally, COPE expression tended to be reduced in both patient and carrier 1 (Figure 3, C and D). A truncated COPA protein could not be detected (Figure 3C). COPA, COPB2, and COPE protein expression in COPA^C1013S^ PBMCs of carrier 3 (C.I.1) and patients 4–6 (C.II.2–4) was normal (Supplemental Figure 3C). Consistent with the findings at the mRNA level, COPA, COPB2, and COPE protein levels were similar in EBV LCLs, derived from patients and carriers of families A and C, and in fibroblasts, of patient 1 (A.II.3), carrier 1 (A.I.1), and patient 2 (B.II.1), compared with controls (Figure 3, E and F).

### COPA mutations impact COPI integrity by disrupting COPI formation (COPA^R1142X^) or COPI stability (COPA^R1058C^ and COPA^C1013S^).

To further evaluate the effect of the mutations on the integrity of COPI, we first generated 3D models of mutant COPA protein structures and evaluated their interaction with COPE (Figure 2D). COPA^R1142X^ was predicted to encode a truncated protein, lacking 84 amino acids of its C-terminal tail. The missing residues include on the one hand the dimerization interface between different COPA-COPE complexes and on the other hand the hydrophobic residues that pack together with α-helices, forming the binding site of COPE. COPA^R1142X^ was predicted to alter the conformation of the entire CTD and subsequently prevent COPA homo-oligomerization and COPE binding (Figure 2D). R1058 and C1013 are located in the α-helices composing the main body of the CTD. COPA^R1058C^ and COPA^C1013S^ likely disrupt the conformation of the α-helices and change the protein’s overall structure. Analysis of COPA^R1058C^ demonstrated that the mutation of the solvent-exposed R1058 to a hydrophobic cysteine presumably affects the stability of COPA. Further, this cysteine could form an unwanted disulfide bond with other free cysteines during protein folding, which is predicted to heavily disrupt the α-helix (Figure 2D). For COPA^C1013S^, the mutation of C1013 to a serine was predicted to decrease the affinity toward the neighboring helix, thus affecting overall protein stability or folding kinetics (Figure 2D). Furthermore, the α-helices, which comprise residues 1013 and 1058, form a binding site for singleton tryptophan motif (STM), a sequence crucial for COPA homo-oligomerization and ER tethering of COPI vesicles (Figure 2E) (39). Therefore, CTD COPA mutations were predicted to affect COPA conformation and to hamper homo-oligomerization, which is essential for COPI integrity.

**Figure 4:**
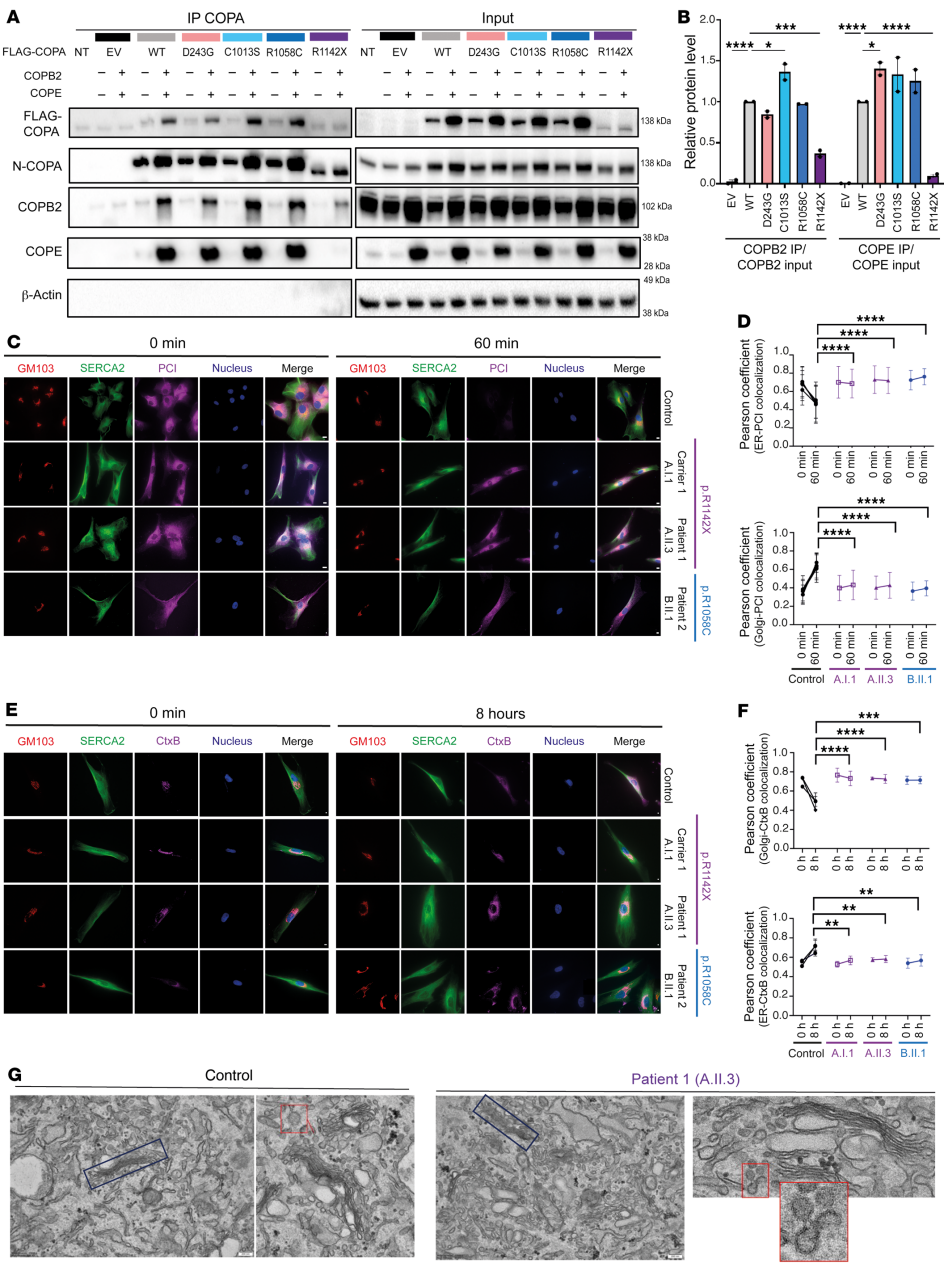
COPA^R1142X^ and COPA^R1058C^ disrupt COPI integrity and impair anterograde ER-to-Golgi and retrograde Golgi-to-ER trafficking. (**A**) Western blot analysis of FLAG, N-terminal COPA, COPB2, COPE, and β-actin antibody in whole-cell extract (input) or eluate of IP of HEK293T cells cotransfected with both WT or mutant COPA and WT or EV of COPB2 and COPE. IP was performed with an antibody against FLAG (IP COPA). (**B**) Quantification of COPB2 and COPE protein levels coimmunoprecipitated with COPA-FLAG (COPA IP) compared with the input signal, as observed in **A**. (**C**) Immunoﬂuorescence analysis of PCI transport assay in 4 different control fibroblasts (1 representative control is shown), COPA^R1142X^ fibroblasts, derived from A.I.1 and A.II.3, and COPA^R1058C^ fibroblasts, derived from B.II.1, at 0 and 60 minutes. Scale bars: 10 μm. (**D**) Graphs represent quantification of the ER exit (top) and Golgi entry (bottom) of PCI, as shown in **C**. (**E**) Immunoﬂuorescence analysis of CtxB transport assay in 4 different control fibroblasts, COPA^R1142X^ fibroblasts, derived from A.I.1 and A.II.3, and COPA^R1058C^ fibroblasts, derived from B.II.1. Analysis was performed 2 hours and 10 hours after exposure to CtxB. Scale bars: 10 μm. (**F**) Graphs represent quantification of the Golgi release (top) and ER entry (bottom) of CtxB, as observed in **E**. In **B**, **D**, and **F**, results are shown as mean ± SEM, and significance levels were calculated using 1-way (**D** and **F**) or 2-way (**B**) ANOVA (*P < 0.05; **P < 0.01; ***P < 0.001; ****P < 0.0001). Data are representative of 2–3 independent experiments. (**G**) Electron microscopy of COPA^R1142X^ fibroblasts, derived from A.II.3, compared with fibroblasts from a healthy control. Original magnification, ×15,000; ×40,000 (bottom right); scale bars: 200 nm. The images demonstrate a fragmented and disorganized Golgi apparatus (blue box) and an accumulation of vesicles (red box) in the cytosol of COPA^R1142X^ fibroblasts.

Next, we evaluated the interaction of mutant COPA with COPB2 and COPE through a coimmunoprecipitation (co-IP) assay. For this purpose, HEK293T cells were transiently transfected with FLAG-tagged WT or mutant COPA, COPB2, and COPE, and co-IP was performed with a FLAG antibody. The ability of mutant COPA to bind COPB2 and COPE was evaluated by immunoblotting of the whole-cell extract, marked as input, and of the IP, for COPA, COPB2, and COPE (Figure 4A). COPA^D243G^ was used as the representative of the WD40 domain mutations. The co-IP assay revealed that while COPA^WT^, COPA^D243G^, COPA^C1013S^, and COPA^R1058C^ were able to pull down COPB2 and COPE, COPA^R1142X^ did not precipitate COPE and displayed a significantly reduced interaction with COPB2, confirming the predicted disruption of COPI formation by COPA^R1142X^ (Figure 4, A and B). Furthermore, immunoblotting using an antibody that detects the FLAG tag revealed a comparable expression of COPA in the input of HEK293T cells transfected with COPA^D243G^, COPA^C1013S^, COPA^R1058C^, and COPA^WT^. In contrast, HEK293T cells transfected with COPA^R1142X^ produced a truncated protein, which displayed a reduced expression compared with the WT or missense mutation COPA proteins, suggesting COPA^R1142X^ instability (Figure 4A).

### Impaired anterograde ER-to-Golgi and retrograde Golgi-to-ER COP-dependent protein trafficking in COPA^R1142X^ and COPA^R1058C^ fibroblasts.

To evaluate the effect of the CTD COPA mutations on COPI function, we examined COPI-mediated intracellular trafficking in COPA^R1142X^ and COPA^R1058C^ fibroblasts. COPA^C1013S^ fibroblasts were unavailable. First, we evaluated the anterograde ER-to-Golgi transport with a procollagen I (PCI) assay, which relies on the temperature-sensitive protein folding of PCI (47). PCI exit from the ER occurs in COPII vesicles and depends on the retrograde recruitment of COPI-coated ERGIC53-containing vesicles (48). The existence of this early COPI-dependent, pre-Golgi cargo sorting step was first demonstrated in mammalian cells and results in a delayed collagen secretion and retention of PCI in the ER in COPB2 siRNA-treated fibroblasts (47, 49). COPA^R1142X^ fibroblasts derived from patient 1 (A.II.3) and carrier 1 (A.I.1), and COPA^R1058C^ derived from patient 2 (B.II.1), demonstrated a delayed ER export to the Golgi of PCI, which was retained at the ER, with higher levels of PCI in the ER for up to 60 minutes after the release of the 40°C temperature block, in line with a disturbed COPI function (Figure 4, C and D). Accumulation of PCI in both COPA^R1142X^ and COPA^R1058C^ fibroblasts was also demonstrated by an increased intensity of PCI 60 minutes after the release of the 40°C temperature block, whereas control cells showed a 50% reduction in their amount of PCI (Supplemental Figure 4). The retention of PCI in mutant cells is most likely due to a defect in secretion.

Second, we performed an assay to measure the retrograde transport of cholera toxin B subunit (CtxB) (50). COPA^R1142X^ fibroblasts derived from patient 1 (A.II.3) and carrier 1 (A.I.1), and COPA^R1058C^ fibroblasts derived from patient 2 (B.II.1), demonstrated a delayed Golgi-to-ER trafficking, with higher levels of CtxB at the Golgi for up to 10 hours after exposure to CtxB (Figure 4, E and F). Next, we evaluated COPA^R1142X^ fibroblasts of patient 1 (A.II.3) by electron microscopy to examine both the morphology of the COPI vesicles and the impact of COPI dysfunction on cellular morphology. As previously described for COPB2 deficient fibroblasts, we found an increased accumulation of coated vesicles in the cytosol and the Golgi apparatus appeared smaller in COPA^R1142X^ fibroblasts in comparison with healthy control fibroblasts (Figure 4G) (47). In conclusion, the impaired ER exit of PCI, the retention of CtxB at the Golgi in COPA^R1142X^ and COPA^R1058C^ fibroblasts, and the disturbed cellular morphology in COPA^R1142X^ fibroblasts demonstrate a disruption of both the anterograde COPII-dependent ER-to-Golgi transport and retrograde COPI-dependent Golgi-to-ER transport caused by COPI dysfunction.

### Type I IFN signaling is increased in 3 of 6 patients with CTD COPA mutations and does not correlate with disease severity.

**Figure 5:**
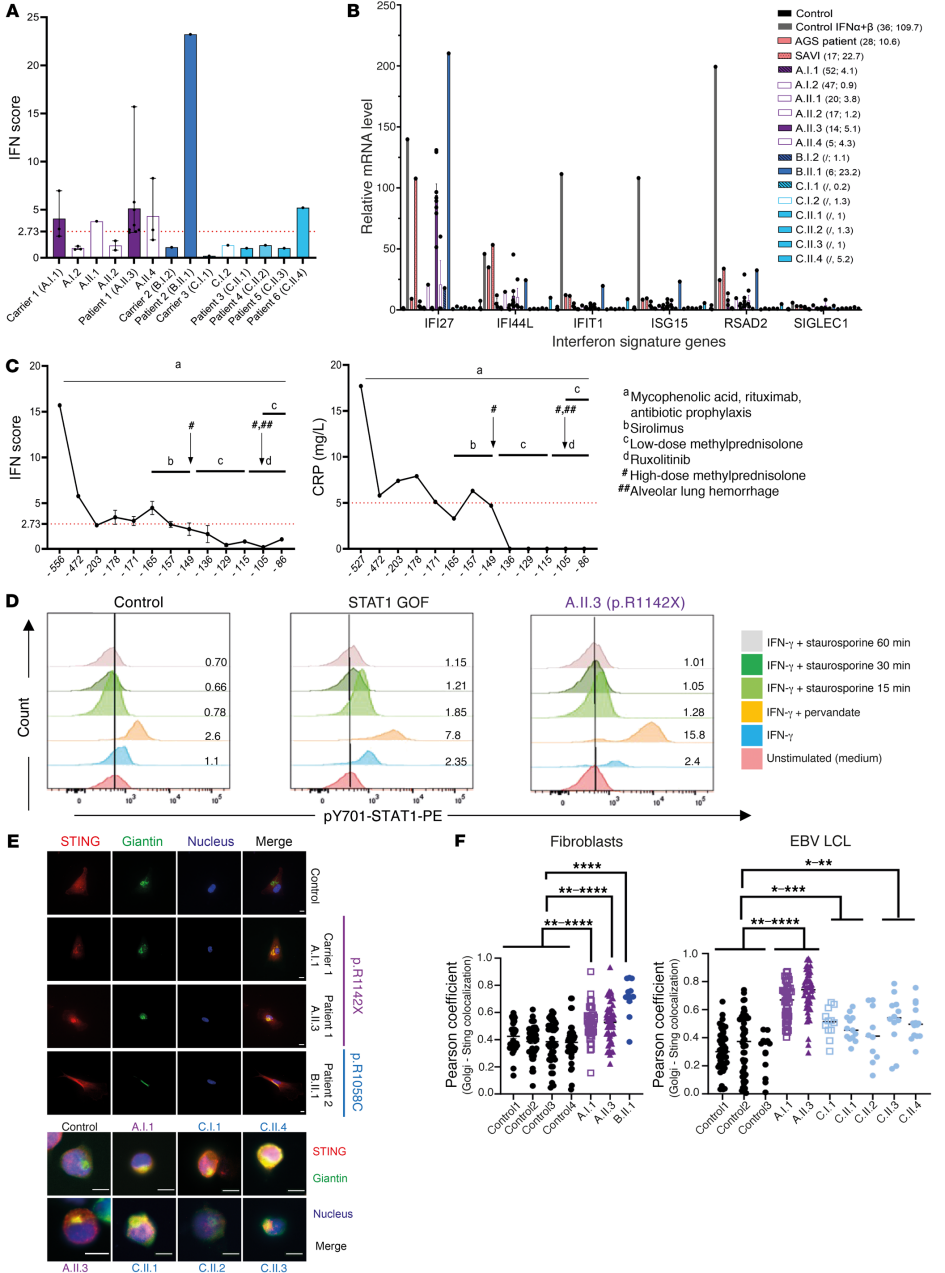
Mutations in the CTD of COPA induce type I IFN pathway activation in 3 of 6 patients. (**A** and **B**) IFN scores (**A**) and ISG expression (**B**) in peripheral whole blood of patients and their families. (**B**) mRNA expression of the individual ISGs for 3 healthy controls, 1 IFN-α– and IFN-β–stimulated PBMC sample of a healthy control, 1 SAVI patient (carrying a p.V155M mutation in STING), 1 AGS patient (heterozygous for a p.S962A fs*92 and a p.P193A mutation in ADAR), and the patients, carriers, and healthy family members included in **A**. The first number in parentheses is the decimalized age at the time of sampling, the second the IFN score. Mean ± SEM values of different time points, if available, are shown (*n* = 7 for A.II.3 [prior to transplantation]; *n* = 3 for A.I.1, A.I.2, A.II.4; *n* = 2 for A.II.2; and *n* = 1 for other individuals). (**C**) Evolution of type I IFN score (left) and CRP value (right) through the disease course of patient A.II.3. Timing is indicated in days prior to hematopoietic stem cell transplantation. Relevant clinical manifestations and treatments are indicated. (**D**) Flow cytometry analysis of p-STAT1 in monocytes of A.II.3 compared with a control and a STAT1 gain-of-function (GOF) patient. The ratio of the number of p-STAT1–positive cells in comparison with the unstimulated condition is indicated. Data are representative of 2 independent experiments. (**E**) Immunoﬂuorescent analysis of STING localization in fibroblasts (top) and EBV LCLs (bottom) of healthy controls, A.I.1, A.II.3, B.II.1, C.I.1, and C.II.1–4. Cells were stained for STING-TMEM173, *cis*-Golgi (giantin), and nucleus (Hoechst). The merge column represents an overlay between the stains. Scale bars: 10 μm. (**F**) Quantification of colocalization of STING and *cis*-Golgi, expressed as the Pearson coefficient. Results are representative of at least 3 independent experiments. Results are shown as means ± SEM, and significance levels were calculated using 1-way ANOVA (*P < 0.05; **P < 0.01; ***P < 0.001; ****P < 0.0001).

Next, we examined whether mutations in the CTD of COPA, analogous to the mutations in the WD40 domain, induce type I IFN signaling. We assessed 6 IFN-stimulated genes (ISGs) by RT-qPCR in RNA extracted from whole blood or PBMCs. In patients 1 (A.II.3), 2 (B.II.1), and 6 (C.II.4) we measured an elevated type I IFN score (Figure 5, A and B) (51). Patients 1 (A.II.3) and 2 (B.II.1), affected by a more severe phenotype, displayed at least on one occasion an elevated type I IFN score within the range seen in STING-associated vasculopathy with onset in infancy (SAVI) or Aicardi-Goutières syndrome (AGS) patients, while patient 6’s (C.II.4’s) score was markedly lower. Although all siblings of family C are symptomatic, only patient 6 (C.II.4) demonstrated an elevated type I IFN score. Asymptomatic carriers and healthy family members demonstrated no type I IFN score or a minimally elevated one. Despite disease progression, serial follow-up of IFN scores of patient 1 (A.II.3), derived from 13 different time points over a period of one-and-a-half years, decreased, in line with the C-reactive protein (CRP) values (Figure 5C) (17). This contrasts with the persistently elevated type I IFN score described in patients affected by WD40 domain COPA mutations. IFN signaling through signal transducer and activator of transcription 1 (STAT1) was shown by ﬂow cytometric evaluation of downstream phosphorylated STAT1 (p-STAT1) in monocytes of patient 1 (A.II.3). Upon blocking of phosphatases and IFN-γ stimulation, there was a significantly higher phosphorylation of STAT1 in patient 1 (A.II.3) compared with both a healthy control and a STAT1 gain-of-function patient, heterozygous for the c.A1159G (p.T387A) mutation in STAT1 (Figure 5D). Further, colocalization demonstrated increased levels of STING in the Golgi of COPA^R1142X^ and COPA^R1058C^ fibroblasts and COPA^R1142X^ and COPA^C1013S^ EBV LCLs compared with controls (Figure 5, E and F). Finally, the ratio of SIGLEC-1–positive monocytes, as a surrogate marker of type I IFN signaling environment, was significantly higher for patient 1 (A.II.3) in comparison with controls (Supplemental Figure 5A). In conclusion, these data demonstrate an upregulation of type I IFN signaling due to CTD COPA mutations in several patients, patient-derived fibroblasts, and EBV LCLs.

### Overexpression of the CTD COPA mutants does not induce STING-dependent type I IFN signaling in HEK293T cells.

**Figure 6:**
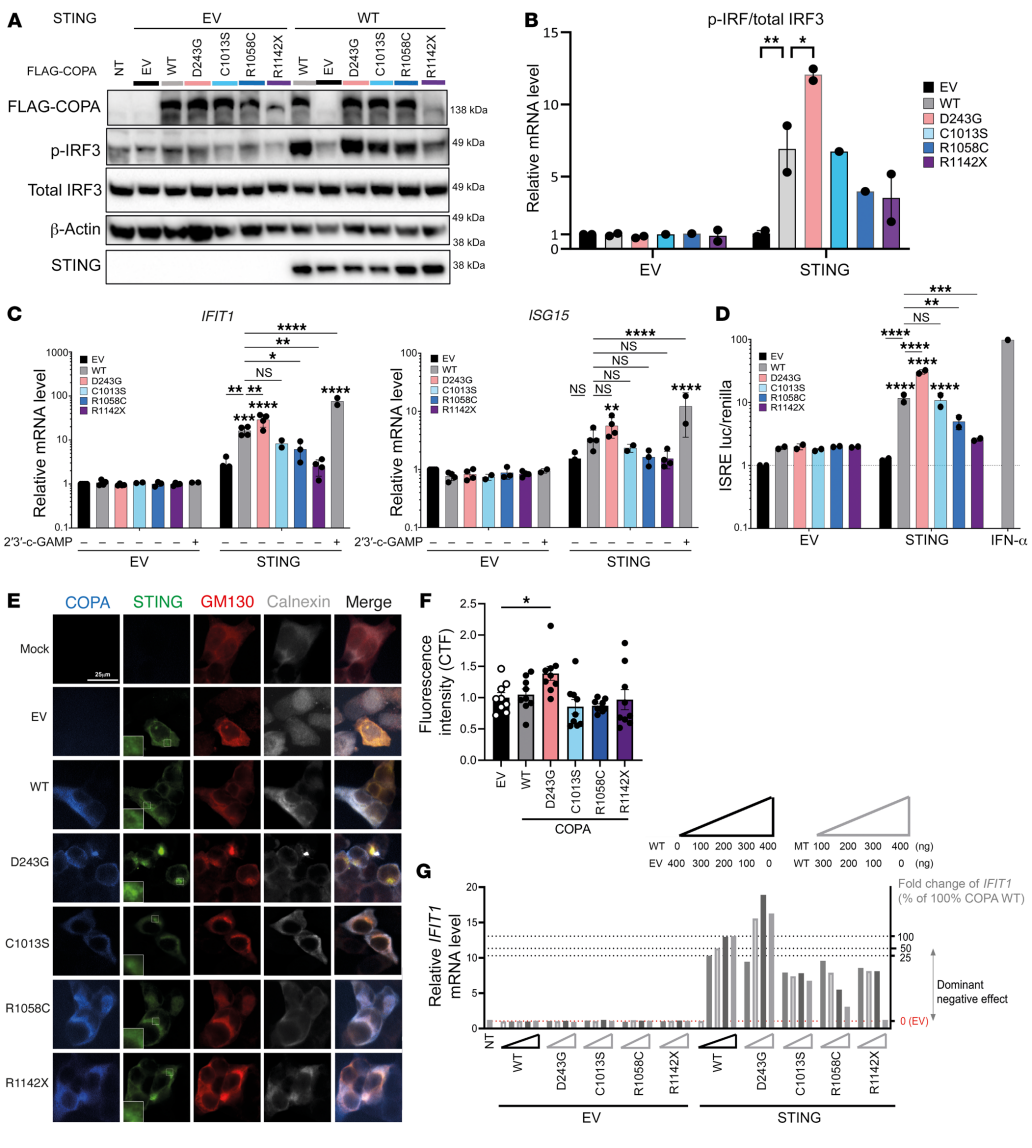
Overexpression of the CTD COPA mutants does not induce STING-dependent type I IFN signaling in HEK293T cells. (**A**–**F**) HEK293T cells were cotransfected with EV or WT STING and WT or mutant COPA as indicated. (**A**) Immunoblotting of whole-cell lysates for COPA (FLAG), p-IRF3, total IRF3, STING, and β-actin. (**B**) Quantification of p-IRF3 protein relative to total IRF3, as demonstrated in **A**. (**C**) Relative mRNA expression of IFIT1 and ISG15, normalized to GAPDH and to HEK293T cells expressing COPA EV and STING EV. First, expression was compared between cells cotransfected with STING and COPA^WT^ and cells cotransfected with STING and mutant COPA (lines and asterisks). Second, expression in cells cotransfected with STING EV and WT or mutant COPA was compared with the corresponding condition cotransfected with STING (asterisks above error bars). Cells stimulated with 2′3′-cGAMP served as a positive control. (**D**) ISRE luciferase reporter assay. Luciferase activity was measured in total cell lysate. (**E**) Confocal microscopy of COPA and STING colocalization. Cells were stained for COPA (FLAG), STING, Golgi (GM130), and ER (calnexin). The additional squares in the STING column contain an enlargement of the image. Scale bar: 25 μm. (**F**) Quantification of the ratio of STING localized to the Golgi over total STING, as demonstrated in **E**. Results in **A**–**F** are representative of 2–4 independent experiments. In **B**–**D** and **F**, columns and bars represent mean ± SEM, analyzed using 1-way (**F**) or 2-way (**B**–**D**) ANOVA (*P < 0.05; **P < 0.01; ***P < 0.001; ****P < 0.0001). (**G**) Relative mRNA expression of IFIT1 in HEK293T cells cotransfected with different ratios of WT and mutant COPA and EV or WT STING. Triangles depict the amount of transfected WT, EV, or mutant COPA cDNA. Dotted lines and the right *y* axis illustrate fold change of IFIT1 corresponding to HEK293T cells cotransfected with different percentages of WT COPA. Variants are classified based on their effect on IFIT1 expression. The mean of 3 technical replicates is shown.

To further study the type I IFN signaling in CTD COPA mutations, HEK293T cells, which endogenously express COPA and lack STING and cGAS, were transiently transfected with STING and WT or mutant COPA. COPA^D243G^ served as the representative of WD40 domain COPA mutations. Western blot analysis of phosphorylated IRF3 (p-IRF3) tended to be reduced in HEK293T cells cotransfected with STING and COPA^R1058C^ or COPA^R1142X^, while HEK293T cells cotransfected with STING and COPA^C1013S^ expressed similar p-IRF3 levels compared with HEK293T cells overexpressing STING and COPA^WT^ (Figure 6, A and B). In contrast, COPA^D243G^ induced significantly increased p-IRF3 levels (Figure 6, A and B). Consistently, IFIT1 mRNA expression was enhanced in HEK293T cells cotransfected with COPA^D243G^ and STING, while HEK293T cells cotransfected with STING and CTD COPA mutants demonstrated a similar, for COPA^C1013S^, or reduced, for COPA^R1058C^ and COPA^R1142X^, level of IFIT1 in comparison with cells overexpressing WT COPA and STING (Figure 6C). Similar differences, yet statistically insignificant, were noted upon evaluation of ISG15 mRNA expression (Figure 6C). An IFN-stimulated response element (ISRE) luciferase assay confirmed a comparable, for COPA^C1013S^, or reduced, for COPA^R1058C^ and COPA^R1142X^, ISRE activation in HEK293T cells cotransfected with CTD mutant COPA and STING in comparison with HEK293T cells overexpressing STING and WT COPA (Figure 6D). Finally, confocal microscopy proved a similar colocalization of STING with Golgi matrix protein 130 (GM130), a peripheral membrane protein of the *cis*-Golgi, in HEK293T cells cotransfected with CTD mutant COPA and STING in comparison with cells overexpressing WT COPA and STING, while an increased colocalization was noticed in HEK293T cells cotransfected with COPA^D243G^ and STING (Figure 6, E and F). To decipher whether the perturbation of STING-dependent type I IFN signaling, caused by CTD COPA mutations, was a dominant-negative or haploinsufficient effect, we evaluated IFIT1 mRNA expression in HEK293T cells cotransfected with, on the one hand, varying ratios of WT COPA, mutant COPA, and empty vector (EV) of COPA and, on the other hand, EV of STING or WT STING (Figure 6G and Supplemental Figure 5B). The variants that reduced the IFIT1 expression below the level corresponding to 50% WT COPA were considered to be dominant negative. We observed a dominant-negative effect of CTD mutant COPA on IFIT1 induction, since the IFIT1 expression in HEK293T cells transfected with CTD COPA mutant, WT COPA, and STING was consistently reduced in comparison with the IFIT1 levels in HEK293T cells overexpressing identical amounts of EV of COPA, WT COPA, and STING (Figure 6G).

In conclusion, overexpression of the CTD COPA mutations in HEK293T cells did not induce increased type I IFN signaling, in contrast with the strong upregulation of type I IFN signaling by COPA^D243G^, pointing to a different mechanism.

### CTD COPA mutations cause activation of ER stress and proinﬂammatory signaling pathways such as the NF-κB pathway.

**Figure 7:**
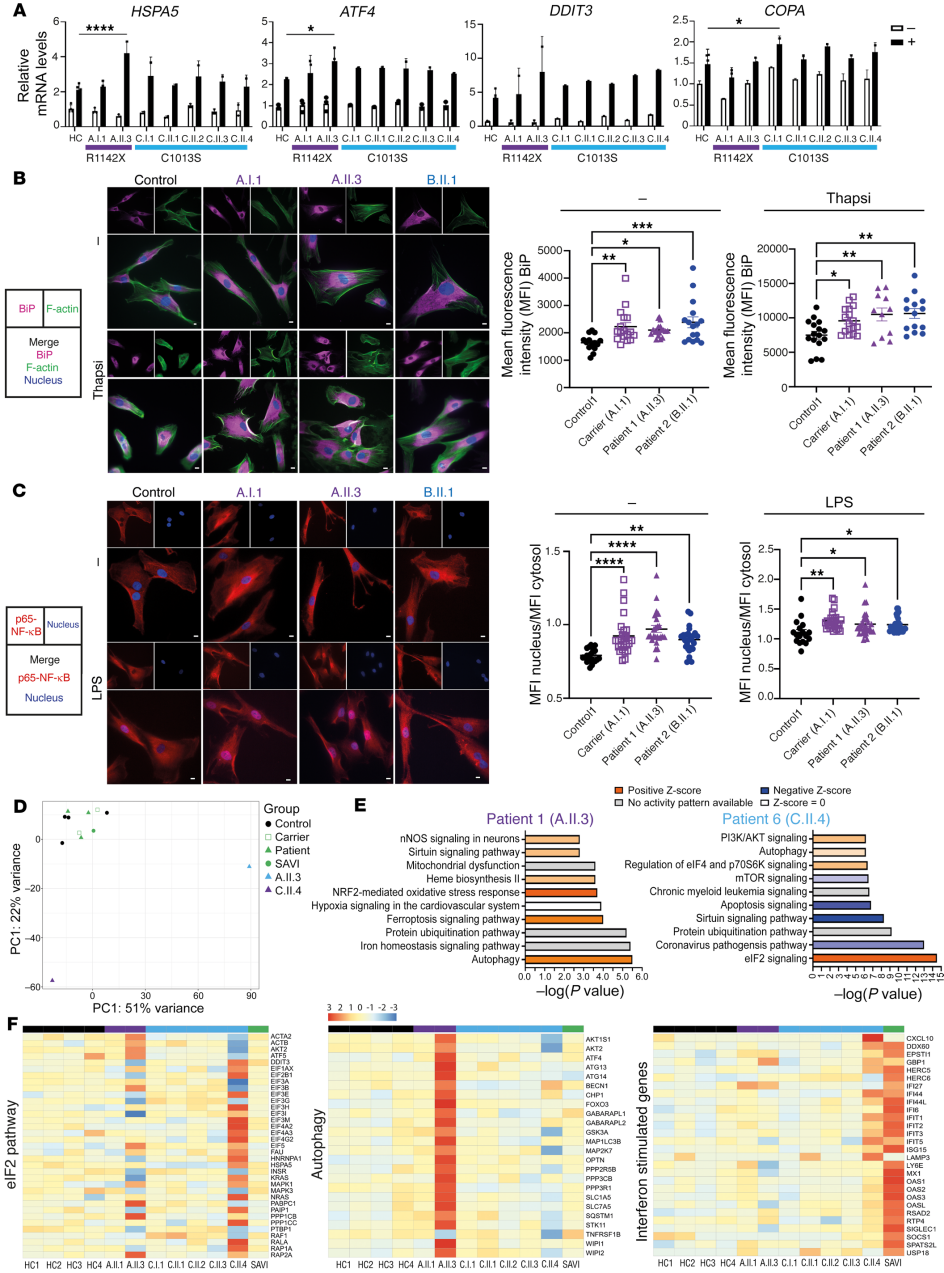
CTD COPA mutations cause activation of ER stress and proinﬂammatory signaling pathways such as the NF-κB pathway. (**A**) Relative mRNA expression of HSPA5, ATF4, DDIT3, and COPA in EBV LCLs of 4 healthy controls, A.I.1, A.II.3, C.I.1, and C.II.1–4. LCLs were unstimulated (white, –) or treated for 6 hours with thapsigargin (black, +). Results were normalized to GAPDH (ΔCt) and to the control samples (ΔΔCt). (**B**) Representative images of immunoﬂuorescent analysis of BiP intensity in fibroblasts of healthy controls, A.I.1, A.II.3, and B.II.1. Cells were stained for BiP, F-actin, and nucleus and stimulated with thapsigargin. Graphs represent quantification of MFI of BiP. (**C**) Representative images of immunoﬂuorescent analysis of p65–NF-κB nuclear translocation in fibroblasts of healthy controls, A.I.1, A.II.3, and B.II.1. Cells were stimulated with LPS and stained for p65–NF-κB and nucleus. Nuclear translocation of p65 appears violet. Graphs represent quantification of nuclear translocation of NF-κB. Scale bars: 10 μm. In **A**–**C**, columns and bars represent mean ± SEM, representative of 2 (**A**) to 3 (**B** and **C**) independent experiments. Statistical analysis was performed using 1-way (**A**) or 2-way ANOVA (**B** and **C**) (*P < 0.05; **P < 0.01; ***P < 0.001; ****P < 0.0001). (**D**–**F**) Analysis of bulk RNA sequencing data of whole-blood RNA of 4 controls (black), 2 carriers (A.I.1 and C.I.1), 1 SAVI patient (green), and 5 patients (A.II.3, C.II.1–4). (**D**) Principal component analysis (PCA) plot of bulk RNA sequencing data, based on the 1,000 genes with the largest intersample variance (after a variance stabilizing transformation removing the variance dependence on the mean). (**E**) Top 10 differentially expressed pathways determined by IPA analysis of differential gene expression for patients A.II.3 (left) and C.II.4 (right) versus the group consisting of carriers (A.I.1, C.I.1), controls, SAVI patient, and C.II.1–3. (**F**) Heatmaps represent differential expression analysis for the eIF2 pathway, 24 autophagy genes, and a limited list of ISGs.

Next we investigated whether dysregulation of other inﬂammatory signaling pathways could contribute to the clinical phenotype and type I IFN signaling observed in patients. We hypothesized that the defective intracellular trafficking caused by CTD COPA mutations increases ER stress and activates the unfolded protein response (UPR), similarly to the effects of mutations in the WD40 domain of COPA (15). Indeed, in COPA^R1142X^, derived from patient 1 (A.II.3) and carrier 1 (A.I.1), and COPA^R1058C^ fibroblasts, derived from patient 2 (B.II.1), we found an increased expression of the molecular chaperone binding immunoglobulin protein (BiP) by confocal microscopy, both in baseline conditions and after stimulation with thapsigargin (Figure 7B). BiP is suggested to act as a primary sensor in the activation of the UPR, which is a stress response activated to restore cellular homeostasis. After treatment with thapsigargin, which induces ER stress by inhibiting the sarco-/endoplasmic reticulum Ca^2+^-ATPase (SERCA), qPCR evaluation of 3 UPR-dependent genes (HSPA5, ATF4, and DDIT3) showed a significantly elevated level of HSPA5 and ATF4 in thapsigargin-treated EBV LCLs of patient 1 (A.II.3) in comparison with controls (Figure 7A). In contrast, COPA^R1142X^ EBV LCLs derived from carrier 1 (A.I.1) did not reveal a statistically significant difference in comparison with controls. COPA^C1013S^ EBV LCLs, derived from patients 3–6 (C.II.3–6) and carrier 3 (C.I.1), demonstrated a tendency toward an increased UPR response upon ER stress induction (Figure 7A). Furthermore, DDIT3 mRNA expression was significantly elevated, for p.C1013S and p.R1142X, or tended to be elevated, for p.R1058C, in unstimulated HEK293T cells overexpressing the CTD COPA mutants in comparison with cells transfected with WT COPA (Supplemental Figure 8).

Secondly, we evaluated the translocation of the transcription factor family nuclear factor-κB (NF-κB), which could potentially be induced by ER stress and could contribute to type I IFN signaling induction. Confocal microscopy in COPA^R1142X^ and COPA^R1058C^ fibroblasts of patients 1 (A.II.3) and 2 (B.II.1) and carrier 1 (A.I.1) was performed to measure the intensity of p65 RelA in the nucleus and cytoplasm (Figure 7C). In unstimulated control fibroblasts, p65 is found almost exclusively in the cytosol, while unstimulated COPA^R1142X^ and COPA^R1058^ fibroblasts demonstrate an increased nuclear presence of p65, indicating a constitutive activation of the NF-κB pathway caused by these CTD COPA mutations. In addition, COPA^R1142X^ and COPA^R1058C^ fibroblasts showed pronounced translocation after 1 hour of stimulation with LPS compared with controls. These findings were confirmed by immunoblotting of nuclear cell extract of TNF-α–stimulated fibroblasts for p65. Fibroblasts of patient 1 (A.II.3) and carrier 1 (A.I.1) showed an increased and prolonged nuclear presence of p65 in comparison with controls (Supplemental Figure 6, A and B).

Finally, we evaluated the mRNA expression of inﬂammatory cytokines in EBV LCLs, at steady state and upon ER stress induction, by qPCR. COPA^R1142X^ EBV LCLs of patient 1 (A.II.3) had increased transcript levels encoding interleukin-1β (IL-1β), in both the unstimulated and the stimulated condition (Supplemental Figure 7). The transcript levels in EBV LCLs derived from carrier 1 (A.I.1), patients 3–6 (C.II.3–6), and carrier 3 (C.I.1) were not statistically significantly upregulated in both conditions. Further, evaluation of the serum cytokine concentrations of patients 1 (A.II.3), 2 (B.II.1), and 3 (C.II.1) demonstrated an increased IFN-γ concentration for patient A.II.3, and increased IL-6, IL-8, and TNF-α concentrations were detected for all patients (Supplemental Figure 5C). These findings point to a complex interplay of various proinﬂammatory signaling pathways.

### Transcriptome analysis by RNA sequencing of peripheral blood reveals an upregulation of the UPR and dysregulation of autophagy in CTD COPA patients.

RNA was extracted from whole blood of 4 healthy controls, 2 CTD COPA mutation carriers (carriers 1 and 3, respectively A.I.1 and C.I.1), 5 COPA patients (patients 1 and 3–6, respectively A.II.3 and C.II.1–4), and 1 SAVI patient, heterozygous for the c.463G>A (p.V155M) variant in STING, as a control. Principal component analysis of the *P* values of significantly enriched genes in the transcriptome demonstrated clustering of the carriers, controls, SAVI patient, and patients 3–6 (C.II.1–3), while patient 1 (A.II.3) and patient 6 (C.II.4) showed a distinct transcriptome profile (Figure 7D). Therefore, patients 1 (A.II.3) and 6 (C.II.4) were compared with the group of carriers, controls, SAVI patient, and patients 3–6 (C.II.1–3) by Ingenuity Pathway Analysis (IPA) of the differential gene expression (Figure 7E). In patient 1 (A.II.3) autophagy was the pathway with the highest significance and likely activated, while in patient 6 (C.II.4) the autophagy pathway was mildly activated. Importantly, the analyzed RNA sample of patient 1 (A.II.3) was obtained 4 months after discontinuation of the treatment with sirolimus, an autophagy inducer. Several genes involved in autophagy showed a strong activation in patient 1 (A.II.3) and a tendency for inhibition in patient 6 (C.II.4) (Figure 7F). In patient 6 (C.II.4), the eukaryotic initiation factor 2 (eIF2) signaling pathway showed the highest statistical significance and appeared activated (Figure 7E). Moreover, in patient 1 (A.II.3) the nuclear factor erythroid 2–related factor 2–mediated (NRF2-mediated) central cytoprotective pathway against oxidative stress was activated (Figure 7E). Patient 1 (A.II.3) and patient 6 (C.II.4) both displayed an activation of the eIF2 signaling pathway (Figure 7F). NRF2 and eIF2 are downstream targets of pancreatic protein kinase R (PKR)–like ER kinase (PERK) and are 2 parallel pathways through which the UPR helps to increase survival in ER-stressed cells (52). While the SAVI patient demonstrated a strong upregulation of several ISGs, patient 6 (C.II.4) showed a milder upregulation, and a clear upregulation was absent for patient 1 (A.II.3), similar to the corresponding type I IFN score at day –86, as shown in Figure 5C (Figure 7F) (53). In conclusion, transcriptomic analysis revealed signatures of elevated ER stress and activated UPR as well as cytoprotective NRF2-regulated genes and activated autophagy.

## Discussion

Here, we report 3 previously undescribed heterozygous mutations, 1 private nonsense and 2 missense, in the CTD of COPA in 6 children affected by a syndrome of autoinﬂammation and autoimmunity (Supplemental Table 5). Two reports mentioned mutations in the CTD of COPA, without functional validation (54, 55). Despite the many similarities with COPA syndrome caused by WD40 domain mutations, including the incomplete penetrance, the severe phenotype unresponsive to multiple lines of treatment in 2 out of 6 patients with CTD mutations is striking. In terms of pathogenesis, we show that COPA^R1142X^ impairs the interaction of COPA with COPB2 and COPE, considering the reduced expression of the truncated COPA^R1142X^ upon overexpression in HEK293T cells. Binding of COPB2 and COPE remained intact for COPA^C1013S^ and COPA^R1058C^ in overexpression, although biomodeling suggested an impaired stability of COPI and a compromised tethering to the NRZ complex as a result of these mutations (40, 56, 57). In line with these findings, the impaired anterograde ER export of PCI and the impaired retrograde transport of CtxB evidence COPI dysfunction, at least in COPA^R1142X^ and COPA^R1058C^ fibroblasts (47, 50). Therefore, our data highlight the importance of the integrity of the CTD of COPA for COPI function and demonstrate the deleterious impact of CTD COPA mutations on COPI function and bidirectional COP-dependent transport of proteins between ER and Golgi (37).

We found an elevated type I IFN score in the peripheral blood of 3 patients and a Golgi accumulation and activation of STING in COPA^R1142X^ and COPA^R1058C^ fibroblasts and COPA^R1142X^ and COPA^C1013S^ EBV LCLs (Figure 5). However, serial evaluation of the type I IFN score of patient 1 (A.II.3) during the disease course demonstrated a poor correlation with disease severity, as previously observed in adenosine deaminase 2 deficiency (58). Furthermore, only one of the patients of family C, patient C.II.4, displayed an elevated IFN score. To gain further insight into the mechanism of type I IFN upregulation, we performed overexpression of the mutants in HEK293T cells. In contrast to mutations in the WD40 domain of COPA, CTD COPA mutations did not induce a STING-dependent type I IFN signaling upon overexpression in HEK293T cells, indicating a distinct mechanism of type I IFN activation (17). We further noticed a differential effect of the CTD COPA mutations on STING-dependent type I IFN signaling (Figure 6), which has previously been observed for WD40 domain COPA mutants, and which seems to reﬂect the severity of the clinical phenotype (17).

We hypothesize that the dominant-negative effect of COPA CTD mutations on ISG expression, demonstrated in the overexpression model, is the result of an impaired intracellular protein transport. In line with this hypothesis, TBK1 phosphorylation and ISG expression are increased in mouse embryonic fibroblasts, mutated in the COPA WD40 domain. However, treatment of these fibroblasts with brefeldin A, an inhibitor of COPI vesicle formation and Golgi-to-ER transport (59), blocks these effects, suggesting that a complete inhibition of the COPI-dependent transport disrupts STING accumulation and activation at the Golgi. Previously, Steiner et al. have demonstrated that the deletion of different COPI subunit proteins, in particular COPA, COPG1, and COPD, induces cGAS-dependent STING activation (36). The authors prove that a defect in retrograde Golgi-to-ER trafficking causes mislocalization of STING to the Golgi. The activation of the type I IFN signaling, however, was dependent on cGAS activation, which was considered to be a consequence of the altered cellular homeostasis in cells affected by COPI dysfunction. Our data demonstrate that the upregulated type I IFN signaling caused by CTD COPA mutants is secondary to an intracellular trafficking defect, and we suggest that induction of type I IFN signaling occurs in a cGAS-dependent manner. cGAS is absent in HEK293T cells, but type I IFN pathway activation in vivo in patients can be driven through cGAS activation as an indirect result of the impaired intracellular trafficking and disturbed cellular homeostasis (36). As suggested by Steiner et al., disrupted cellular homeostasis could cause a secondary activation of cGAS by leakage of mitochondrial DNA into the cytoplasm, ER stress–induced zinc release in the cytoplasm, or accumulation of genomic self DNA in the cytoplasm (36).

Autoinﬂammation in patients with CTD COPA mutations likely results from the activation of multiple proinﬂammatory signaling pathways, next to the type I IFN pathway. First, ER stress plays a complex role in the induction of inﬂammation (60). The impairment of the retrograde Golgi-to-ER transport caused by mutations in different COPI subunit proteins was previously described to result in an increased ER stress in activated T and/or B cells, heterozygous for mutations in the WD40 domain of COPA or homozygous for mutations in COPG1, or in unstimulated patient-derived fibroblasts, heterozygous for mutations in COPD or homozygous for mutations in COPB2 (47, 61–63). Similarly, we observed an increased ER stress in COPA^R1142X^ and COPA^R1058C^ fibroblasts and an upregulated UPR in activated EBV LCLs of COPA^R1142X^ of patient 1. In COPA^C1013S^ EBV LCLs this effect was less pronounced, although we observed a tendency toward an activation of the UPR. These data were supported by transcriptome analysis by RNA sequencing of peripheral blood of patients 1 (A.II.3) and 6 (C.II.4). A different pattern of UPR gene activation was noted in both patients, which could reﬂect a continuum between adaptive, in patient 6 (C.II.4), and maladaptive UPR response, in patient 1 (A.II.3), as well as the effects of different treatments (64). We hypothesize that the increased ER stress and activation of the UPR could result from an impaired retrieval of chaperone proteins to the ER, due to a limited retrograde Golgi-to-ER retrograde transport, and the accumulation of nascent proteins in the ER, caused by an impaired ER-to-Golgi anterograde transport. This hypothesis is supported by the upregulation of DDIT3 observed in HEK293T cells overexpressing the CTD COPA mutants. Increased ER stress and UPR upregulation are therefore key pathogenic mechanisms caused by CTD COPA mutants. Although diverse inborn errors of immunity underlie increased ER stress and UPR upregulation, the pathways leading to increased ER stress differ, and this may at least in part contribute to the differential phenotypical expression.

Secondly, COPA^R1142X^ and COPA^R1058C^ fibroblasts demonstrate an activation of the NF-κB pathway, and EBV LCLs of patient 1 (A.II.3) showed increased expression of IL-1β. Additionally, the concentration of NF-κB–related cytokines, such as IL-6 and TNF-α, was increased in the serum of patients 1, 2, and 3 (A.II.3, B.II.1, and C.II.1). In contrast, the concentration of IL-6, IL-10, and TNF-α was reportedly normal in the plasma of patients affected by mutations in the WD40 domain of COPA (17). Finally, transcriptome analysis of peripheral blood has revealed a dysregulation of autophagy in patients 1 (A.II.3) and 6 (C.II.4), as previously demonstrated in vitro for mutations in the WD40 domain of COPA (15). COPI is indeed known to be obligatory for autophagy (65).

There are some limitations to this study. Although the CTD COPA mutations studied here display a common disease-causing mechanism, the clinical and the cellular phenotype are heterogeneous, like for WD40 domain COPA mutations. As for the discrepancy between the ubiquitous expression of COPA and the mostly immunological phenotype, we can only hypothesize that the strong expression of COPA in monocytes plays a role and that our understanding of the phenotype will continue to evolve with the description of additional patients.

In summary, mutations in the CTD of COPA, a previously unstudied domain, cause autoinﬂammation through a mechanism distinct from the N-terminal domain COPA mutations. The molecular mechanism, however, shows striking similarities with previously described coatopathies. We demonstrate that mutations in the CTD of COPA impair COPI integrity, which causes a disruption of COPII-dependent anterograde ER-to-Golgi and COPI-dependent retrograde Golgi-to-ER transport, at least in COPA^R1142X^ and COPA^R1058C^ fibroblasts. In our patients, we provide evidence for a concerted activation of several proinﬂammatory pathways, including ER stress and the UPR as well as NF-κB signaling next to type I IFN signaling. Our report therefore expands the phenotype and genotype of COPA syndrome and highlights the crucial importance of the integrity of COPI and the COPI-dependent transport between Golgi and ER in intracellular homeostasis. Our findings pave the way for the discovery of mutations in molecules that affect the COPI-dependent transport between Golgi and ER and underlie yet undetermined inborn errors of immunity.

## Methods

### Patients and sample collection.

Patients were recruited from the Inborn Errors of Immunity Department, University Hospitals Leuven (Leuven, Belgium); the Pediatric Immunology and Rheumatology Department, St. Louis Children’s Hospital (St. Louis, Missouri, USA); and the Pediatric Immunology, Allergy and Retrovirology Department, Texas Children’s Hospital (Houston, Texas, USA). Clinical and laboratory data were collected for all included patients. If possible, parents and siblings of the index case were also included. Blood samples were collected from individual members of family A (A.I.1, A.I.2, A.II.1–4), family B (B.I.2 and B.II.1), and family C (C.I.1, C.I.2, C.II.1–4). EBV LCLs were created from whole blood of A.I.1, A.II.3, C.I.1, and C.II.1–4. The severe clinical condition and heavily immunocompromised condition of patient B.II.1 hindered a blood sample collection for creation of EBV LCLs. A skin biopsy, to create a fibroblast cell line, could be performed in patient A.II.3, carrier A.I.1, and patient B.II.1. Unfortunately, no skin biopsy could be performed in members of family C.

### Whole-exome sequencing and Sanger sequencing.

Whole-exome sequencing (WES) was performed for members of family A (A.I.1, A.I.2, A.II.3, and A.II.4), family B (B.I.2 and B.II.1), and the siblings of family C (C.II.1–3) except for C.II.4. Sanger sequencing was performed to confirm the identified COPA variants. The methods used for genomic DNA preparation, WES analysis, and Sanger sequencing are described in Supplemental Methods.

### Patients’ cells, (primary) immortalized cell lines, and cell culture.

Isolation of PBMCs from the blood of patients and healthy donors, creation of primary immortalized cell lines (EBV LCLs and fibroblasts), and cell culture were performed according to standard methods, as described in Supplemental Methods.

### RNA extraction and RT-qPCR.

The methods used for mRNA isolation and cDNA preparation and RT-qPCR analysis are explained in detail in Supplemental Methods. A list of the probes is supplied as well (Supplemental Table 6).

### Western blotting.

Immunoblotting was performed on cell lysates using standard methods as described in Supplemental Methods; antibodies are listed in Supplemental Table 7.

### Plasmids.

The creation and usage of plasmids for overexpression in HEK293T cells are described in detail in Supplemental Methods.

### Cell transfection.

HEK293T cells (ATCC) were seeded in a 24-well plate, a 96-well plate (luciferase assay), or a T25 ﬂask (co-IP), in DMEM supplemented with 10% FCS and transfected at 70% conﬂuence. First, for cotransfection of COPA and STING, WT or mutant COPA pCMV6-AN-DDK DNA plasmids were cotransfected with a pMSCV vector encoding STING or EV of STING (17). Two hundred nanograms of each DNA plasmid was employed using X-tremeGENE 9 DNA Transfection Reagent (Merck). When different ratios of WT and mutant COPA cDNA were cotransfected, the total amount of COPA cDNA was maintained at 400 ng. To demonstrate the dominant-negative effect, the fold change of 400 ng of WT COPA cotransfected with STING was considered as 100%; of 300 ng of WT COPA and 100 ng of EV of COPA as 75%; of 200 ng of WT COPA and 200 ng of EV of COPA as 50%; of 100 ng of WT COPA and 300 ng of EV of COPA as 25%; and of 400 ng of EV of COPA as 0%. After 24 hours, cells were stimulated with 2′3′-cGAMP. Forty-eight hours after cotransfection, cells were harvested and whole-cell lysate was prepared or Trizol (500 μL per well) was added for RNA extraction. Secondly, for the co-IP assay, 1,800 ng of each plasmid was cotransfected using Lipofectamine 2000 (Invivogen). WT or mutant COPA pCMV6-AN-DDK DNA plasmids were cotransfected with a pCMV6-XL5 and pCMV6-AC plasmid encoding, respectively, COPB2 and COPE or their EVs.

### Coimmunoprecipitation.

Forty-eight hours after transfection, cells were harvested and whole-cell extract was prepared as described above. Extracts were incubated overnight with 1 μg of anti-DDK (FLAG) monoclonal antibody (Origene) (Supplemental Table 7). The following day, Protein G Magnetic Beads (Surebeads, Bio-Rad) were added. After 1 hour of incubation, the beads were magnetized using a DynaMag-2 magnet (Invitrogen, Thermo Fisher Scientific) and washed several times with PBS 0.1% Tween-20. Bolt LDS Sample Buffer (4×, Novex) was used as an elution buffer, and finally denaturation was performed at 95°C for 3 minutes. Concurrent with the immunoblotting of the obtained eluate, a tenth of the amount of the whole-cell extract used for the IP was blotted as the input condition. Immunoblotting for FLAG antibody, COPB2, and COPE was performed as described above (Supplemental Table 7).

### Structural modeling.

Structural modeling of COPA and its interaction with COPE was performed as described in Supplemental Methods.

### Trafficking assays.

All cells were deposited on Lab-Tek Chamber Slides (154534, Thermo Fisher Scientific) and cultured at 37°C, 5% CO_2_ in a humid environment. For the evaluation of the anterograde ER-to-Golgi transport, we followed endogenous PCI in fibroblasts. Fibroblasts were incubated for 3 hours at 40°C in DMEM supplemented with 10% FCS, antibiotics (penicillin and streptomycin), and sodium pyruvate, and then shifted to 32°C for the indicated time. For the evaluation of the retrograde transport (CtxB assay), Alexa Fluor 555–labeled cholera toxin B subunit (CtxB) was obtained from Molecular Probes (C34776). Cells were incubated with 0.15 μg/mL CtxB on ice for 30 minutes. After washing twice with PBS, the cells were incubated with prewarmed DMEM for the indicated times (time 0 corresponds to 2 hours after exposure to the cholera toxin; time 8 hours thus corresponds to 10 hours of incubation with the cholera toxin). Cells were fixed with 4% paraformaldehyde (Electron Microscopy Sciences) for 10 minutes. Then they were washed once in saturation buffer (PBS 1% BSA [MilliporeSigma]), permeabilized with Triton 0.1% buffer for 10 minutes, and washed twice in permeabilization buffer (PBS containing 0.1% saponin [Fluka Biochemika] and 0.2% BSA). Cells were then incubated for 45 minutes with the following primary antibodies based on the type of experiment: anti-PCI, anti-GM130, goat anti-SERCA2 ATPase antibody (Supplemental Table 8). Cells were then washed twice with 1.5 mL of saponin buffer to remove nonspecifically bound antibodies and incubated with secondary antibodies for 30 minutes. Cells were washed twice in saponin buffer and twice with 500 μL of PBS. The labeling of the nuclei was next performed with 1 μg/mL of Hoechst for 5 minutes in the dark. Cells were finally washed twice with 1.5 mL PBS, and mounted with FluorSave Reagent (Millipore). All experiments are representative of at least 3 independent experiments.

### Intracellular ﬂuorescent labeling, confocal microscopy, and image analysis.

Intracellular ﬂuorescent labeling in HEK293T cells, EBV LCLs, and fibroblasts and quantification of the results were performed as described in Supplemental Methods.

### Electron microscopy.

Electron microscopy was performed on fibroblasts of a healthy control and patient A.II.3 as detailed in Supplemental Methods.

### IFN score in patient samples.

To evaluate the IFN score, the mRNA expression of 6 ISGs (*IFI27*, *IFI44L*, *IFIT1*, *ISG15*, *RSAD2*, and *SIGLEC1*) was evaluated by RT-qPCR analysis of cDNA, extracted from peripheral blood or PBMCs as described above. The mRNA expression of these ISGs was normalized to the expression level of GAPDH. As previously described, the mRNA expression of each of the 6 ISGs was expressed relative to a single calibrator (51). The median fold change of the 6 genes compared with the median of 20 previously collected healthy controls was used to create an IFN score for each individual, with an abnormal score defined as >2 SDs above the mean of the control group, i.e., 2.73. For individual A.II.3, several cDNA samples were available and calculation of IFN scores through the disease course was possible.

### Cytokine evaluation in serum samples.

Cytokine evaluation in serum was performed as described in Supplemental Methods.

### Flow cytometry.

Flow cytometry methods used for the evaluation of p-STAT1 in PBMCs and CD169/SIGLEC-1 in monocytes and the Th cell staining are described in detail in Supplemental Methods. The antibodies used are listed in Supplemental Methods and Supplemental Table 7.

### Luciferase assay.

HEK293T cells were seeded into a 96-well plate and cotransfected as described above with 200 ng of WT or mutant COPA and 200 ng of EV or WT STING. Additionally, a luciferase reporter plasmid (100 ng; Promega) and a Renilla Luciferase Control Reporter Vector (20 ng; Promega) were cotransfected. After 24 hours a positive control was generated through stimulation with IFN-α (10,000 U/mL; Life Technologies). Thirty-six hours after transfection, the luciferase activity in the total cell lysate was measured.

### Evaluation of ER stress in EBV LCLs and fibroblasts.

As previously described, baseline and induced ER stress were evaluated in EBV LCLs (14). ER stress was induced experimentally by stimulation with thapsigargin, an inhibitor of the ER Ca^2+^ ATPase. One million cells/mL were treated either with DMSO, in the unstimulated condition, or with thapsigargin (10 μM; MilliporeSigma), in the stimulated condition, during 6 hours. Subsequently the mRNA expression of 3 UPR genes (DDIT3, HSPA5, and ATF4) in unstimulated and stimulated conditions was evaluated. For each gene, Ct values were normalized to GAPDH and secondly to the mean Ct value of the unstimulated healthy control samples. To evaluate BiP intensity in fibroblasts by confocal microscopy, cells were stimulated for 20 hours with thapsigargin (10 μM; Thapsi, MilliporeSigma), and fixation, permeabilization, and intracellular ﬂuorescent labeling were performed as described in Supplemental Methods.

### NF-κB nuclear translocation in fibroblasts.

Evaluation of NF-κB nuclear translocation in fibroblasts by immunoblotting and confocal microscopy was performed as described in Supplemental Methods.

### Bulk RNA sequencing.

Bulk RNA sequencing was performed on RNA isolated from PAX tubes (BD Biosciences, catalog 762165). A detailed description is available in Supplemental Methods.

### Web resources.

The URLs for data presented in this paper, as well as programs used, are as follows: Chromas: https://technelysium.com.au/wp/; Prism: www.graphpad.com; Picard: http://broadinstitute.github.io/picard/; Annovar: https://annovar.openbioinformatics.org/en/latest/; UniProt: https://www.uniprot.org/; BioRender: www.biorender.com

### Statistics.

All data are presented as mean ± SEM or mean ± SD, as indicated in the figure legends. Statistical significance for single comparisons was calculated using the 1-way ANOVA or 2-way ANOVA, and mixed-effects analysis was used to examine repeated measures. Dunnett’s, Šidák’s, and Tukey’s multiple-comparison tests were used to control for multiple comparisons. Statistical significance for multiple comparisons was computed as specified in the figure legends. Analyses were performed with Prism software (version 9.0.0 for Windows, GraphPad Software) as indicated in the figure legends. *P* values less than 0.05 were considered significant.

### Study approval.

This study was approved by the Ethics Committee Research UZ/KU Leuven (protocol S65153, S63078). All participating individuals or their legal representatives gave written informed consent prior to sample collection and processing. Informed consent was in accordance with the Declaration of Helsinki.

### Data availability.

The RNA sequencing data were deposited to the Sequence Read Archive database under Bioproject ID PRJNA1051072. Source data are provided in the Supporting Data Values file. Reported patients did not consent to share their genetic data on a public database. Additional data can be obtained upon request to the corresponding author.

## Author contributions

SD, LM, JD, and IM designed this research study. IM, LM, and JD supervised. IM, GB, and SD provided funding. SD, AI, LM and TMB conducted experiments and LE and RT provided technical assistance. SD, AI, JD, and IM wrote the manuscript. AV and BM provided biomolecular models. MCP analyzed data. DL and BB generated RNA sequencing data. MB and SD performed RNA sequencing analysis. SM provided access to the electron microscopy facility. PB and KV performed electron microscopy. AL, MLF, XB, RS, DR, and MCP provided resources and edited the paper IM, CMD, TMB, MAC, RR, GB, KJ, DM, IC, CC, and PD participated in clinical care. AC, KW, and WH performed genetic analysis and generated primary cell lines. All authors edited the paper.

## Supplementary Material

Supplemental data

Supporting data values

## Version 1 01/04/2024

In-Press Preview

## Version 2 02/15/2024

Electronic publication
