# Supplementary material for: Heterozygous mutations in the C-terminal domain of COPA underlie a complex autoinﬂammatory syndrome
Source: J Clin Invest. 2024 Jan 4;134(4):e163604. doi: 10.1172/JCI163604 (PMC10866661; doi:10.1172/JCI163604)
Supplement: Supplemental data [file jci-134-163604-s191.pdf]

Supplemental Tables

PATIENTS		1 (A.II.3)	2 (B.II.1)	3 (C.II.1)	4 (C.II.2)	5 (C.II.3)	6 (C.II.4)
COPA mutation		WT/p.R1142X	WT/p.R1058C	WT/p.C1013S	WT/p.C1013S	WT/p.C1013S	WT/p.C1013S
Demographic characteristics and presenting symptoms	Etnicity	Caucasian	African American	Caucasian	Caucasian	Caucasian	Caucasian
	Gender	F	M	M	F	M	M
	Birth	Term	Term	Term	Term	Preterm (29w PMA)	Preterm (29w PMA)
	Age at onset (years)	8	5	5	2	1	0
	Initial presentation	Neuromyelitis optica	Livedo reticularis, acrocyanosis, heat sensitivity, increased somnolence and unusual sleep-wake cycle	Abdominal pain, nausea, vomiting and purpura	Gait disturbances, lower extremity weakness, and fever	Recurrent URTI	Hemoptysis
	Status (current age in years)	Alive (16)	Deceased (7)	Alive (19)	Alive (18)	Alive (8)	Alive (8)
Phenotype	**LUNG**						
	Isolated lung cysts	No	No	Yes	No	No	No
	Interstitial lung disease	Yes	Yes	No	No	No	No
	Alveolar hemorraghe	Yes	Recurrent	No	No	No	Yes
	**NEUROLOGIC**						
	Dysautonomia	No	Yes (bradycardia, hypothermia, sensory axonal polyneuropathy)	No	Yes (complex regional pain syndrome)	Yes(insidious onset left hand weakness, secondary urinary incontinence)	No
	Myelitis transversa	Yes	No	No	No	No	No
	Neuromyelitis optica	Yes (recurrent)	No	No	No	No	No
	ASD	Yes	No	No	No	No	No
	Neurodegenerative phenotype	Yes	No	No	No	No	No
	RENAL						
	IgA nephropathy	Chronic nephritis	No	Yes	Chronic nephritis	No	No
	Renal biopsy	No	No	25% crescents and diffuse mesangiocapillary proliferation with immunofluorescence staining positive for IgA	No	No	No
	**INFLAMMATION**						
	Recurrent sterile fever	Yes	No	No	No	Yes	No
	MAS	No	Yes (MOF)	No	No	No	No
	JOINT						
	Arthralgia (isolated)	Yes (legs)	No	Yes (bilateral knee)	Yes (bilateral hand)	Yes(chronic nocturnal thoraco-lumbar back pain)	Yes (hip and knee)
	Rheumatoid) arthritis	No	No	No	No	No	No
	Avascular necrosis	No	No	No	No	No	No
	**SUSCEPTIBILITY TO INFECTIONS**						
	Viral	Recurrent (URTI)	No	No	No	Recurrent (URTI)	Recurrent (URTI)
	Bacterial	H. influenzae	Yes (bullous impetigo)	No	No	Yes(pneumonia)	No
	Fungal	No	Yes (respiratory)	No	No	No	No
	**CUTANEOUS**						
	Livedo reticularis	Yes	Yes	No	Vasculitis	No	No
	Necrotic skin lesions	No	No	No	No	No	No
	Raynaud's phenomenon/acrocyanosis	No	Acrocyanosis	No	No	No	No
	**METABOLIC**						
	Osteopenia	Yes	No	No	No	No	No
	**ONCOLOGIC**	No	No	No	Neuroblastoma	No	No
Immunological laboratory results	Cytopenia	Pancytopenia	Pancytopenia	No	No	No	No
	Hypercytosis	No	Thrombocytosis	No	No	No	No
	Hypo-/hypergammaglobulinemia	Hypogammaglobulinemia	No	Hypogammaglobulinemia	No	No	No
	ESR (mm/h) (maximum value)	27	95	9	8	53	2
	Ferritin (µg/L) (maximum value)	797	221	NA	NA	18	NA
Immunomodulatory treatments		IVIG, Mycophenolic acid, Rituximab, Rapamycin, Ruxolitinib, HSCT	IVIG, Steroids, Rituximab, Azathioprine, Cyclophosphamide, Ruxolitinib, Anakinra, Emapalumab	IVIG, Steroids, Rituximab, Azathioprine	No	No	No

Supplemental Table 1. **Demographic and clinical characteristics of patient A.II.3, B.II.1, C.II.1-4.** IVIG, intravenous immunoglobulin; URTI, upper respiratory tract infections; PMA, post menstrual age; MAS, macrophage activation syndrome; MOF, multiple organ failure, HSCT, hematopoietic stem cell transplantation; ASD, autism spectrum disorder; H. influenza, Haemophilus influenzae.

Patients		1 (A.II.3)	2 (B.II.1)	3 (C.II.1)	4 (C.II.2)	5 (C.II.3)	6 (C.II.4)
**SERUM**	ANA	1:80***	-	1:160***	NA	-	NA
	ANCA	-	+	-	NA	-	NA
	p-ANCA	-	+	-	NA	-	NA
	RF	-	-	-	NA	-	NA
	MPO	-	+**	-	NA	-	NA
	Anti-OJ	-	+*	-	NA	-	NA
	SSB	-	-	-	NA	-	NA
	RNP	-	-	-	NA	-	NA
	Sm	-	-	-	NA	-	NA
	SSA	-	-	-	NA	-	NA
	SSB	-	-	-	NA	-	NA
	Scl-70	-	-	-	NA	-	NA
	Jo-1	-	-	-	NA	-	NA
	Ds-DNA	-	-	-	NA	-	NA
	HLA-B27	-	-	-	NA	-	NA
	Anti-aquaporine-4 antibodies	+	NA	NA	NA	NA	NA
	Anti-cerebrum antibodies	-	NA	NA	NA	NA	NA
	Anti-cerebellum antibodies	+	NA	NA	NA	NA	NA
	Anti-NMO antibodies	+	NA	NA	NA	NA	NA
**CEREBROSPINAL FLUID**	Anti-aquaporine-4 antibodies	+	NA	NA	NA	NA	NA

Supplemental Table 2**. Autoantibodies detected in serum or cerebrospinal fluid of patient A.II.3, B.II.1, C.II.1-4**. + or - refers to positive or negative test result. NA,not available; *, normal levels of muscle enzymes and normal strength upon clinical examination; **, initially positive, second sample negative; ***, speckled, nucleolar.

Patient		1 (A.II.3)		2 (B.II.1)		3 (C.II.1)	4 (C.II.2)	5 (C.II.3)
Therapy		Prior to Rituximab	Rituximab treatment*	Prior to Rituximab	Rituximab treatment*	Prior to Rituximab	NA	NA
Age at the time of sample		8 y.o.	9 - 12 y.o.	6 y.o.	6 y.o.	6 y.o.	14 y.o.	4 y.o.
**Immunoglobulins**	Age related reference values							
IgG (g/l)	4,62 - 16,82	12,90	6,89	8,12	6,83	2,30	9,92	7,58
IgA (g/l)	0,34 - 2,74	0,60	0,19	1,27	2,04	3,43	1,91	0,79
IgM (g/l)	0,30 - 2,51	1,29	0,36	0,35	0,27	0,54	0,42	1,42
**Immune cell subsets**								
Absolute lymphocyte count (n x 10^9 cells/L)	1,66 - 3,45	1,96	1,73	3,24	0,22	2,24	1,67	2,88
CD3 T-cells (%)	63,20 - 77,80	79,80	96,70	59,00	97,00	73,00	86,70	67,00
CD3 T-cells absolute (n x 10^9 cells/L)	1,24 - 2,61	1,56	1,67	1,90	2,14	1,64	1,44	1,93
CD4 T-cells (% of T cells)	31,70 - 47,00	46,80	45,50	45,00	66,00	27,70	48,80	30,40
CD4 T-cells absolute (n x 10^9 cells/L)	0,65 - 1,52	0,92	0,79	1,46	1,47	0,62	0,81	0,78
CD8 T-cells (% of T cells)	17,10 - 30,00	31,50	48,30	13,00	29,00	40,00	32,00	27,10
CD8 T-cells absolute (n x 10^9 cells/L)	0,37 - 0,95	0,62	0,87	0,42	0,64	8,98	0,53	0,78
CD4/CD8 ratio	1,18 - 2,65	1,49	0,94	3,50	1,00	0,69	1,53	1,12
CD56+ T-cells(% of CD3 T cells)	5,40 - 18,60	3,80	3,60	NA	NA	1,00	4,20	12,00
HLA-DR+ T-cells(% of CD3 T cells)	2,30 - 7,00	11,90	51,90	1,00	1,00	18,00	NA	6,00
CD27+CD45RA+ T-cells (% of CD3 T cells)	53,30 - 74,00	54,80	20,70	NA	NA	18,00	19,20	20,00
CD3+ CD4-CD8- T-cells (% of CD3 T cells)	6,00 - 14,60	4,80	2,40	NA	NA	2,00	1,4	1,80
CD19 B-cells (%)	12,00 – 24,00	9,70	0,30	33,00	0,00	0,50	8,00	18,70
CD19 B-cells absolute (n x 10^9 cells/L)	0,27 - 0,64	0,19	0,01	1,07	<0,27	0,11	0,13	0,49
CD27+ IgM+IgD+ (% of B-cells)	3,00 - 21,10	10,90	/	NA	/	3.70	6,50	1,40
CD27+ IgM-IgD- (% of B-cells)	4,00 - 20,50	4,20	/	NA	/	74.10	9,40	1,30
CD27- IgM+IgD+ (% of B-cells)	58,50 - 48,60	77,20	/	NA	/	NA	67,7	90,60
CD16, CD56 NK cells (%)	7,00 - 12,00%	5,10	3,50	5,00	2,00	14,00	4,60	12,00
CD16, CD56 NK cells absolute(n x 10^9 cells/L)	0.07 - 1.20	0,01	0,06	1,62	<0,35	0,31	0,08	0,36

Supplemental Table 3. **Immunophenotyping and immunoglobulin levels of patient A.II.3, B.II.1 and C.II.1-3.** Immunophenotyping data at 2 different time points during disease course are reported for patient A.II.3 and B.II.2, including indication of the corresponding treatment**.** Immunophenotyping data of patient 6 (C.II.4) were unavailable. *, prior to IVIG treatment; NA, not applicable.

**Family**	**A**	**B**	**C**
**Genomic position**	160260473	160261695	160261911
**Chromosome**	1	1	1
**cDNA change**	c.3424C>T	c.3172C>T	c.3038G>C
**Amino acid change**	p.R1142X	p.R1058C	p.C1013S
**Polyphen-2**	NA	Probably damaging	Tolerable
**SIFT**	NA	Damaging	Tolerable
**CADD**	48	35	22
**AF**	Private	0.000017	0.000010
**Zygosity**	Heterozygous	Heterozygous	Heterozygous
**Ref SNP cluster ID**	NA	rs772182530	rs775980693

Supplemental Table 4. **Genetic characteristics and evaluation by *in silico* prediction tools of pathogenicity of CTD *COPA* mutations.** Genomic position according to the hg19(GRCh37) physical position. NM_004371.4 was used as reference transcript. CADD, Combined Annotation-Dependent Depletion; Polyphen: Polymorphism Phenotyping v2; AF, Allele Frequency; SIFT, Sorting Intolerant From Tolerant; NA, not applicable.

	Mutation	**COPA$^{R1142X/WT}$**	**COPA$^{R1058C/WT}$**	**COPA$^{C1013S/WT}$**			
	Patients	**(A.II.3)**<br>**1**	**(B.II.1)**<br>**2**	**(C.II.1)**<br>**3**	**(C.II.2)**<br>**4**	**(C.II.3)**<br>**5**	**(C.II.4)**<br>**6**
Phenotype severity		Life-threatening	Fatal	Less severe			
mRNA expression<br>*(whole blood)*	*COPA*	↓	Tendency ↓	Tendency ↓			
	COPB2	↓		↓	Tende ncy ↓	↓	Tendency ↓
	COPE	Tendency ↓		Tendency ↓			
Protein expression<br>*(PBMC)*	COPA	N-COPA tendency ↓, C-COPA ↓	NA	=			
	COPB2	↓					
	COPE	Tendency ↓					
mRNA and protein expression<br>*(EBV LCL, fibroblast)*	COPA, COPB2, COPE	= *(EBV LCL, fibroblast)*	= *(Fibroblast)*	= *(EBV LCL)*			
COPI integrity	Biomodelling	COPI disintegrity	Stability ↓	Stability ↓			
	Co-IP (HEK293T)	COPI disintegrity (x COPE, ↓COPB2)*	=	=			
COPI function	PCI assay (fibroblast)	↓ anterograde ER-Golgi PCI transport		NA			
	CtxB assay (fibroblast)	↓ retrograde Golgi-ER CtxB transport		NA			
	EM (fibroblast)	Disturbed	NA				
ype I IFN signaling	Whole blood, PBMC	↑	↑	=	=	=	↑

	Patient-derived cell line	↑ *(EBV LCL, fibroblast)*	↑ *(Fibroblast)*	↑ *(EBV LCL)*			
	HEK293T	↓↓	↓	=			
Pro-inflammatory signalling pathways, other than type I IFN signaling	ER stress (RNA seq**, EBV LCL, fibroblast)	↑ (RNA seq, *EBV LCL, fibroblast)*	↑ *(EBV LCL)*	Tendency ↑ *(EBV LCL)*		↑ (RNA seq, *EBV LCL)*	
	NF-κB (fibroblast)	↑	↑	NA			
	Cytokines (EBV LCL, serum)	↑ (IL-1β, IL-6, IL-8, IFN-γ, TNF-α)	↑(IL-6, IL-8, TNF-α)	↑(IL-6, IL-8, IL-10, TNF-α)	NA		
	Autophagy (RNA seq**)	Activated	NA	=	=	=	Activated

Supplemental Table 5. **Summary of research findings concerning the study of mutations in the CTD of COPA.** Patients of families A, B and C with indication of the genotype, assigned with the amino acid changes. ↓, significantly reduced in comparison to control condition; tendency ↓, tendency to be reduced in in comparison to control condition; ↑, upregulated in comparison to control condition; PCI, type I procollagen; CtxB: Cholera toxin B subunit; EM, electron microscopy; PBMC, peripheral blood mononuclear cells; EBV LCL, EBV-transformed lymphoblastoid B-cells; N-COPA, immunoblotting with antibody specific for N-terminal region of COPA; C-COPA, immunoblotting with antibody specific for C-terminal region of COPA; RNA seq**, RNA sequencing performed on whole blood of patients. NA, not available; =, not significantly different compared to control condition; *, impaired precipitation of COPE and significantly reduced binding of COPB2.

**gDNA COPA PCR**	**Type**	**Primer**	**Sequence**
	M13tag	COPAR1142X_F	5'-TGTAAA ACGACGGCCAGT TGTCTCACATCAACGGAAC-3'
		COPAR1142X_R	5'-TAGATCCTATAACTCGCTTCCGTCCTTTGTCGATACTGG-3'

**cDNA COPA PCR**	**Type**	**Forward primer (5'-3')**	**Reverse primer (5'-3')**
COPA_c1	SYBR Green	TGTAAAACGACGGCCAGTTCGGAGAC CTGAGAGATGTT	AACCATCATAGTCGGTCGGAGTCCTTTGTCGATACTGG
COPA_c2	SYBR Green	TGTAAAACGACGGCCAGTTTTCTGGGTC CTAGCTGCTC	GAAGCTCTACGCCTGAGATAGTCCTTTGTCGATACTGG
COPA_c3	SYBR Green	TGTAAAACGACGGCCAGTGCCTCCGATG ATCAGACCAT	ACACAAATTCGACCTTGCCCGTCCTTTGTCGATACTGG
COPA_c4	SYBR Green	TGTAAAACGACGGCCAGTCTGTCCTAGA TCGGATGCATT	TCCATGACGTGTACCACTCCGTCCTTTGTCGATACTGG
COPA_c5	SYBR Green	TGTAAAACGACGGCCAGTTCAAGCTGGC CCTGATCAAC	GATTTCCTCTGTAAACTGGGGTCCTTTGTCGATACTGG
COPA_c6	SYBR Green	TGTAAAACGACGGCCAGTCTATCTCACA GCTGCTACCCA	ACTGGTTCATCCCCATTAGGTGTCCTTTGTCGATAC TGG
COPA_c7	SYBR Green	TGTAAA ACGACGGCC AGTTGCGGCTCCTTCATGACCA	CTGAGATCTTGTCGTCTTCGGTCCTTTGTCGATACTGG
COPA_c8	SYBR Green	TGTAAAACGACGGCCAGTCAAGAGATTG CAGAGGCCCA	ATTGTCGTGGAAGGGGGACGTGTCCTTTGTCGATACTGG

**COPA mutagenesis**	**Variation**	**Forward primer (5'-3')**	**Reverse primer (5'-3')**
	p.D243G	TGGGAGGTTGgTACCTGCCGG	CCGCGTACTTACTTAGTTTCCGT
	p.C101C3S	TGGCAGCTGTCCTACCAGCTC	GAATTACTGGAGTAGGTTGCC
	p.R1058C	CACCATTTGCTGTGAGTACATTGTG	TCTCCGGGTCGTCGAGTA
	p.R1142X	CCAACAGACCTGAAAAATCCTGTCTGC	CGGGTTCGGACTCCACCG

**cDNA qPCR**	**Primer**	**Forward primer (5'-3')**	**Reverse primer (5'-3')**
	COPA_1	GCTTTCACCCCAAAAGACCTT	GCTCCGTAACTGAAGGTATTC
	COPA_2	ATTTGTGCTGCATCATATCGG	TCACTGTCTCTAACCGTTTC
	COPA_3	CGCTGTCTTTTCACATTGCT	GCTCACACCTTGACCGTTAGA
	COPA_4	ATGCTGTTCATGGCAATATGCT	CAACGCCTCACCAAGGTTC
	COPB2	CTAATCTGCATGTCATCCGCTC	AGAAGTGGTCACAGACACAAAG
	COPE	AGGCGCTAGACAAGGATAGTGG	AGGTCCGGCACGCGTACAAACG
	STING	TACTACTCCCTCCCAAATGCGGTC	GTAGCCTATAGACGCCGACTA
	HSPA5	CAAGCAACCAAAGACGCTGGA	CTCCACTGTTTGGCTATGGACA
	ATF4	GTTCTCCAGCGACAAGGCTA	CTACGGGACAACCCATATCT
	DDIT3	AGAGGAAGAATCAAAAATCT	AGGTCTAAGGTCAGTCTCGA
	IL-1β	GATCACTGAACTGCACGCTCC	TCCTATACCTCGTTGTTCAC
	IFNα-2a	GAGTTTGGCAACCAGTTCCA	TTCCTGAGTAGACGACGAAC
	IFI27	CTCTGCCGTAGTTTTGCCCCT	ACACTAACCTCCTCAACACCGG
	IFI44L	AGTTTAATTCCCGTAAACCA	CAACCGTTTTCACTTCGTTC
	IFIT1	ATGAGTACAAATGGTGATGA	CAGAACCTAGTCTAACTTAA
	ISG15	GGTGGACAAATGCGACGAACCTC	ACTCGCCCGACCTCCCACAC
	SIGLEC1	ACAACTTCCGCTTCGAGATCAGT	AGAGCTCCCGTGTCTCCACCT
	GAPDH	GTCTCCTCTGACTTCAACAGCG	AACCGATGTCGTTGTCCCACCA

Supplemental Table 6. **PCR and RT-qPCR primers used in this study.**

	Antibody	Host species	Source	Catalog reference
**Western blot and colocalization**				
	STING D2P2F	Rabbit monoclonal	Cell Signaling	#13647
**Western blot**	Anti-alpha COP I/COPA antibody - C-terminal	Rabbit polyclonal	Abcam	ab192919
	Anti-alpha COP I/COPA antibody - N-terminal	Rabbit monoclonal	Abcam	ab181224
	Anti-COPB2	Rabbit polyclonal	Abcam	ab229639
	Anti-COPE	Rabbit polyclonal	Abcam	ab235061
	Anti-DDK OTI4C5(Flag)	Mouse monoclonal	OriGene	TA50011-1
	Phospho-(Ser396)IRF3	Rabbit monoclonal	Cell Signaling	#4947S
	IRF3	Rabbit monoclonal	Cell Signaling	#11904
	NF-κB p65	Rabbit monoclonal	Cell Signaling	#8242
	Anti-lamin B	Mouse monoclonal	Santa Cruz Biotechnology	AB_2861346
	Goat anti-rabbit IgG H&L (HRP)	Goat polyclonal	Abcam	ab205718
	Goat anti-mouse IgG HRP(secondary antibody) Conjugate	Goat	Novagen	71045-3
	β-actin (secondary antibody)	Rabbit monoclonal	Cell Signaling	#3700
**Flowcytometry**	p-STAT1 PE	Mouse monoclonal	Biolegend	612564
	CD14 APC-eF780	Mouse monoclonal	eBioscience	47-0149-42
	CD3 APC-eF780	Mouse monoclonal	eBioscience	47-0036-42
	CD4 PeCy7	Mouse monoclonal	BD Biosciences	348809
	CD45 RA FITC	Mouse monoclonal	BD Biosciences	335039
	CXCR5 AF647	Rat monoclonal	BD Biosciences	558113
	CCR6 BV421 (CD196)	Mouse monoclonal	BD Biosciences	562515
	CXCR3 PE (CD183)	Mouse monoclonal	BD Biosciences	560928
	CD14 FITC	Mouse monoclonal	BD Biosciences	345784
	CD16 BV421	Mouse monoclonal	BD Biosciences	562874
	Anti-Human Lineage Cocktail (CD3, CD19, CD20, CD56; APC)	Mouse	Biolegend	363601
	HLA DR PerCP-eF 710	Mouse monoclonal	BD Biosciences	46-9952-42
	Pe IgG1 κ Isotype Control (CD169)	Mouse monoclonal	BD Biosciences	555749
	Anti-Human SIGLEC-1 (CD169 Pe)	Mouse monoclonal	BD Biosciences	565248

Supplemental Table 7. **List of antibodies used in this study.**

	Antibody	Host species	Source	Catalog reference
**Microscopy**	Anti-PC-I	Rabbit polyclonal	Rockland	600-401-103S
	Anti-GM130	Mouse monoclonal	BD Biosciences	610822
	Anti-GM130	Rabbit polyclonal	ThermoFisher	# PA1-077-A555
	Anti-SERCA2 ATPase	Goat polyclonal	Abcam	Ab219173
	Calnexin	Mouse monoclonal	ThermoFisher	#MA3-027
	Anti-Flag (M2)	Mouse monoclonal	Sigma-Aldrich	F3165
	Anti-coatomer CM1A10	Mouse monoclonal	Gift from R. Venditti	Not available
	Anti-Giantin	Rabbit polyclonal	Abcam	ab80864
	Anti-TGN46	Rabbit polyclonal	Abcam	ab50595
	Human STING-TMEM173	Mouse monoclonal	R&D Systems	#723505
	Anti-p65-NF-kB	Mouse monoclonal	Santa Cruz Biotechnology	sc-8008
	anti-GRP78 BiP	Rabbit polyclonal	abcam	ab21685
	Anti-STING D2P2F	Rabbit monoclonal	Cell Signaling	#13647c
	Donkey anti mouse IgG-Alexa 647		Invitrogen	A32787
	Donkey anti rabbit IgG-Alexa 568		Invitrogen	A10042
	Donkey anti goat IgG-Alexa 488		Invitrogen	A11055
	Donkey anti rabbit IgG-Alexa 488		Invitrogen	A21206

Supplemental Table 8. **List of antibodies for microscopy in this study.**

Supplemental Figures

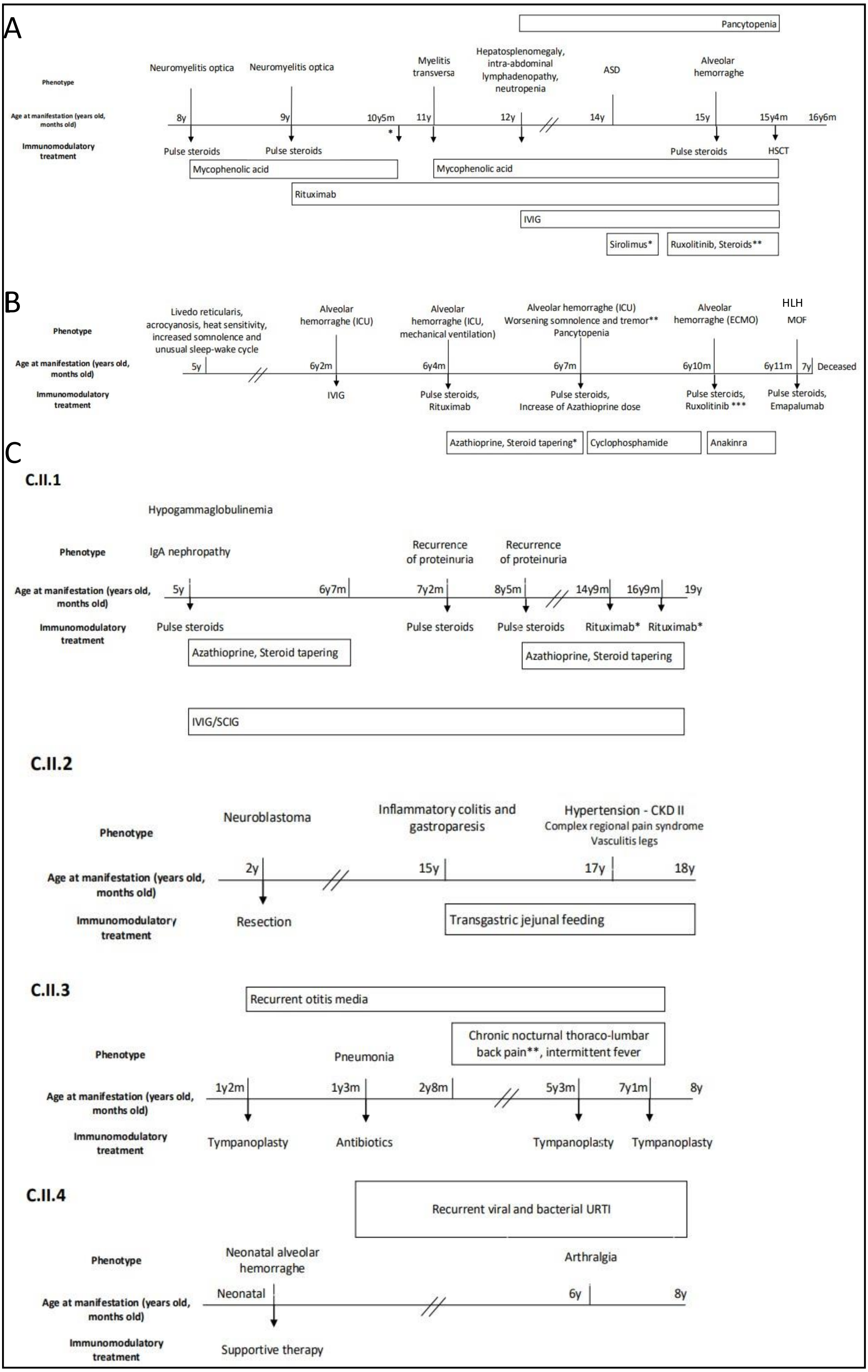

Supplemental Figure 1. **Clinical course of patient 1 (A.II.3), 2 (B.II.1), 3-6 (C.II.1-4). (A)** Clinical course of patient 1 (A.II.3). Intravenous immunoglobulin (IVIG); hematopoietic stem cell transplantation (HSCT); autism spectrum disorder (ASD); *, stopped due to side effects; **, significant clinical improvement since steroid therapy however relapse upon steroid tapering. **(B)** Clinical course of patient 2 (B.II.1). Hemophagocytic lymphohistiocytosis (HLH); multiple organ failure (MOF); *, significant clinical improvement since steroid therapy however relapse upon steroid tapering; **, cerebral MRI showed mild focal narrowing in circle of Willis of unclear etiology; ***, stopped due to side effects (hypertriglyceridemia with secondary pancreatitis). **(C)** Clinical course of patients 3-6 (C.II.1-4). Subcutaneous Immunoglobulin (SCIG); *, significant improvement of proteinuria since start therapy, **, resolution after treatment with non-steroidal anti-inflammatory drugs.

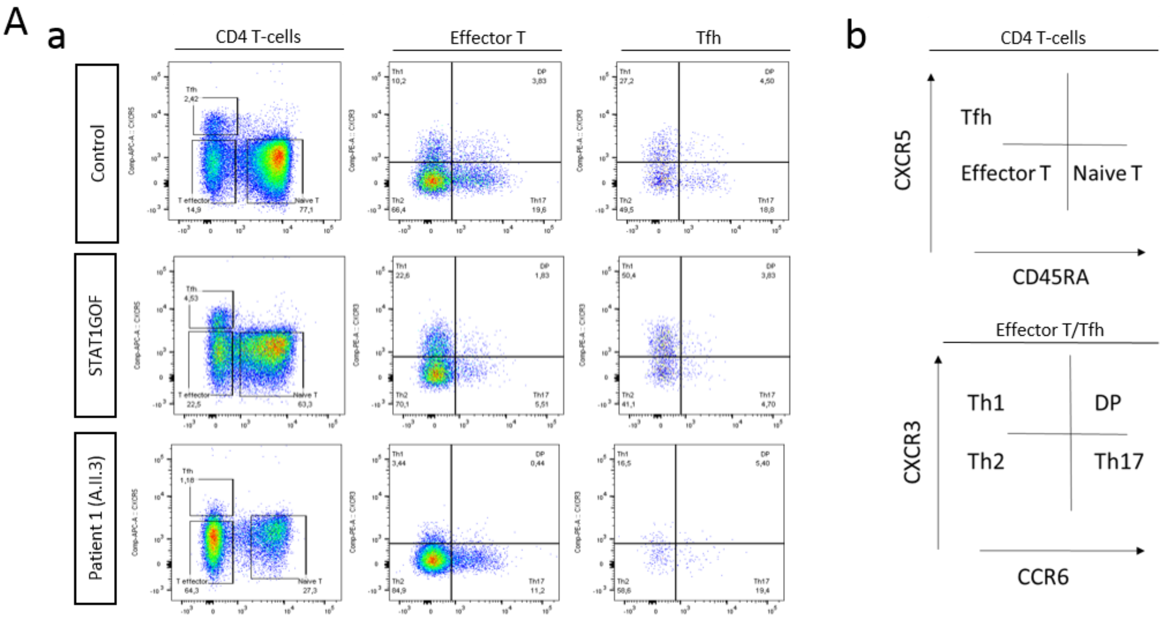

c

	CD4 T-cells			Effector T			T follicular helper		
	Naive T	Effector T	Tfh	Th1	Th2	Th17	Th1	Th2	Th17
Control	77,1%	14,9%	2,4%	10,2%	66,4%	19,6%	27,2%	49,5%	18,8%
STAT1GOF	63,3%	22,5%	4,5%	22,6%	70,1%	5,51%	50,4%	41,1%	4,7%
Patient 1 (A.II.3)	27,3%	64,3	1,2%	3,4%	84,9%	11,2%	16,5%	58,6%	19,4%

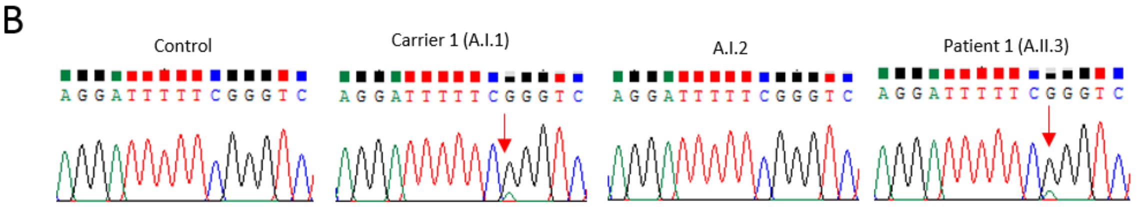

Supplemental Figure 2. **T helper cell immunophenotyping and COPA cDNA sequencing in whole blood of patient 1 (A.II.3). (A)** T helper cell immunophenotyping of patient 1 (A.II.3), a STAT1 gain-of-function (GOF) patient, heterozygous for the c.A1159G (p.T387A) mutation in *STAT1*, compared with a healthy control. Scatterplots demonstrate flow cytometry data and gating strategy for T helper cell subpopulations in subpanel a and b. Subpanel c shows the relative amount in percentages of the different T-helper cell subpopulations. Tfh, T follicular helper cell; DP, double-positive T cell; Th1, Type 1 T helper; Th2, Type 2 T helper, Th17, Type 17 T helper. **(B)** Sanger sequencing chromatograms of *COPA* cDNA extracted from whole blood of a control, carrier 1 (A.I.1), individual A.I.2 and patient 1 (A.II.3) are demonstrated. Sanger sequencing is shown covering a 15 bp snapshot around the mutation. Red arrows indicate the position of the mutation.

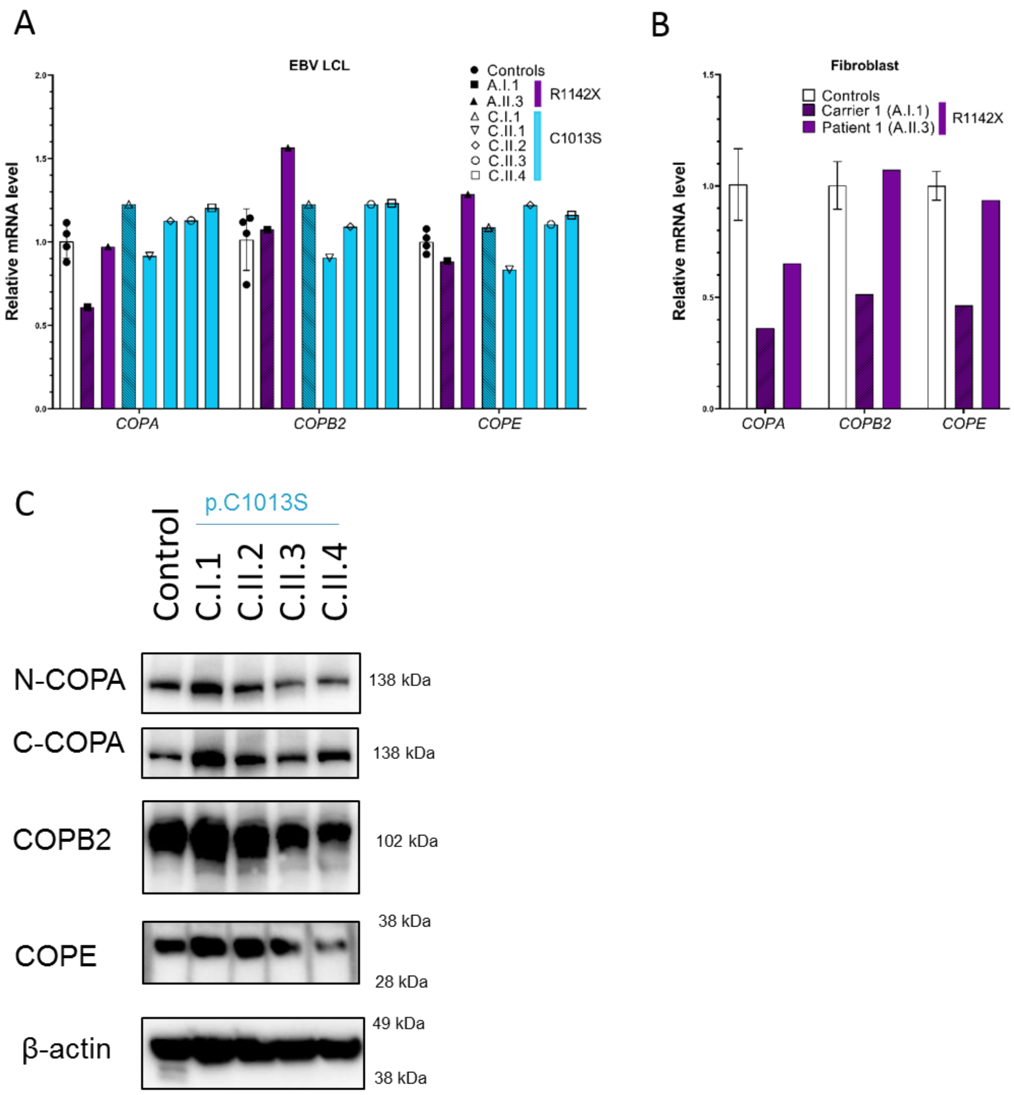

Supplemental Figure 3**. mRNA expression of COPA, COPB2 and COPE in EBV LCLs and fibroblasts and protein expression of COPA, COPB2 and COPE in COPA^{C1013S} PBMC.** **(A)** RT-qPCR analysis of transcript levels of *COPA* (calculated as the mean of the transcript levels of the 4 COPA probes, not shown), *COPB2* and *COPE* in cDNA extracted from EBV LCLs of patients and carriers, from family A (A.I.1, A.II.3) and C (C.I.1, C.II.1-4), in comparison to controls. The relative mRNA level depicts the fold increase of the expression of the gene of interest normalized to GAPDH (ΔCT) and to the mean ΔCT of the control samples. Relative mRNA levels from patient and carriers were compared with the mean of 4 healthy controls. Mean ± SEM values are shown, statistically analyzed using two-way ANOVA and the Dunnett's multiple comparisons tests (not significant values are not depicted). **(B)** RT-qPCR analysis of transcript levels of *COPA* (calculated as the mean of the transcript levels of the 4 COPA probes, not shown), *COPB2* and *COPE* in cDNA extracted from fibroblasts derived from carrier A.I.1, patient A.II.3 in comparison to controls. The relative mRNA level depicts the fold increase of the expression of the gene of interest normalized to GAPDH (ΔCT) and to the mean ΔCT of the control samples. Relative mRNA levels from patient and carriers were compared with the mean of 4 healthy controls. Mean ± SEM values are shown, statistically analyzed using two-way ANOVA and the Dunnett's multiple comparisons tests (not significant values are not depicted). **(C)**Western blot analysis of COPA, COPB2 and COPE in whole cell lysates of PBMCs of a healthy control, C.I.1 and C.II.2-4. Immunoblotting of COPA was performed with an antibody specific for the N-terminal region of COPA (N-COPA) and an Ab specific for the C-terminal region of COPA (C-COPA).

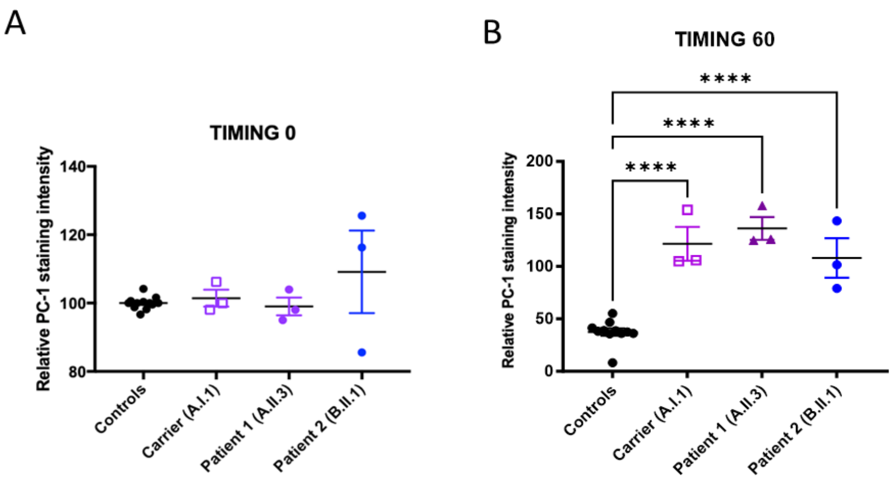

Supplemental Figure 4. **Relative PCI staining intensity.** Analysis of type I proto-collagen (PCI) intensity following anterograde transport. Graph representing 3 independent experiments. Each point in the graph corresponds to the average of an experiment. The data were normalized by the average of the controls at timing 0. A) Intensity of the staining at timing 0. No difference in expression of PC1 is observed when comparing the 4 different controls with the patients. B) Intensity of PCI marking after 60 minutes. The amount of PCI was increased for patients and the carrier and decreased in 4 different controls, suggesting a specific block in the secretion of PCI only in patients' fibroblasts.

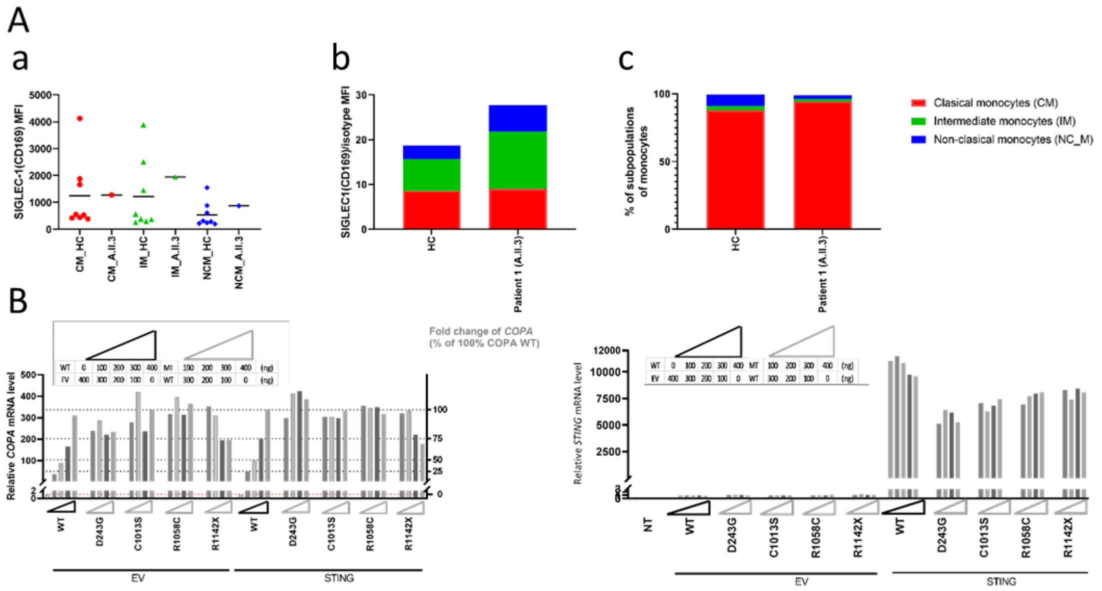

Supplemental Figure 5. **(A)** Flow cytometry data for evaluation of monocytes, monocyte subpopulations and the expression of CD169/SIGLEC-1, analyzed in PBMCs of patient 1 (A.II.3) and 8 healthy controls. The dot plot in subpanel a demonstrates the mean fluorescence intensity (MFI) of Anti-Human SIGLEC-1 (CD169) in classical (CD14+CD16−), intermediate (CD14+CD16+) and non-classical (CD14−CD16+) monocytes. No statistical difference could be observed upon comparison of the mean of each subpopulation in the healthy controls versus the patients by ordinary one-way ANOVA and Šídák's multiple comparisons test. Subpanel b demonstrates the MFI ratio of Anti-Human SIGLEC-1 (CD169) relative to Pe IgG1 κ Isotype Control (CD169) positive monocytes and their subtypes. Subpanel c indicates the frequencies of each monocyte subpopulation. CM, classical monocytes; IM, intermediate monocytes; NC_M, non-classical monocytes. **(B)** qPCR analysis of mRNA expression of *COPA* (left panel) and *STING* (right panel) in HEK293T cells co-transfected with different ratios of WT COPA and mutant COPA and EV of STING or STING. The triangle depicts the amount of WT, EV or mutant COPA cDNA transfected in HEK293T cells. The dashed lines and the right y-axis illustrate the fold change of COPA corresponding to HEK293T cells co-transfected with different amounts of WT COPA, expressed in percentages. The fold change of 400ng of WT COPA co-transfected with STING was considered as 100%, 300ng of WT COPA and 100ng of EV of COPA as 75%, 200ng of WT COPA and 200ng of EV of COPA as 50% and 100ng of WT COPA and 300ng of EV of COPA as 25% and 400ng of EV of COPA as 0%. Experiment was independently performed once.

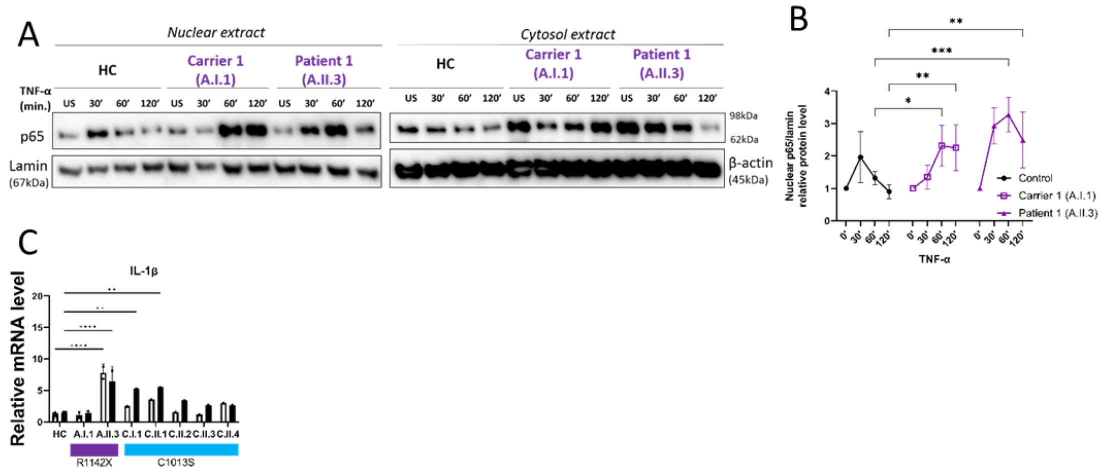

Supplemental Figure 6. **Activation of NF-κB pathway caused by COPA pR1142X mutation** (**A)** Immunoblotting of p65 in nuclear (left panel) and cytoplasmic (right panel) cell extract of fibroblasts of a healthy control, carrier 1 (A.I.1) and patient 1 (A.II.3). Fibroblasts were treated for the indicated time with TNF-α. Data are representative for 3 independent experiments. **(B)** Quantification of nuclear protein amount of p65 normalized to lamin and to the unstimulated condition as observed in B. Mean ± SEM values of three independent experiments are shown, statistically analyzed using two-way ANOVA and the Dunnett's multiple comparison tests. (*, P<0,05; **, P<0,01; not significant values are not indicated on the graph). **(C)** qPCR analysis of *IL1-β* in unstimulated and thapsigargin treated EBV LCLs of 4 healthy controls, carrier 1 (A.I.1), patient 1 (A.II.3), carrier 3 (C.I.1) and patients 3-6 (C.II.1-4). EBV LCLs were unstimulated (white; -) or treated for 6 hours with thapsigargin (black; +). Results were normalized to *GAPDH* (ΔCT) and to the mean ΔCT of the control samples (ΔΔCT). Data from two independent experiments are included.

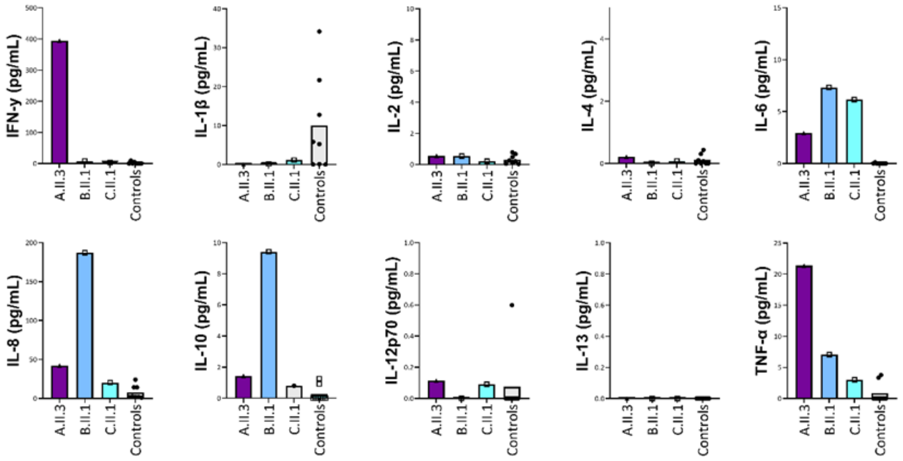

Supplemental Figure 7**. Concentration of pro-inflammatory cytokines in serum of COPA patients.** MSD V-plex analysis of the concentration (pg/ml) of pro-inflammatory cytokines IFN-γ, IL-1β, IL-2, IL-4, IL-6, IL-8, IL-10, IL-12p70, IL-13 and TFN-α in serum of controls (six adults, two children) and patients 1 (A.II.3), 2 (B.II.1) and 3 (C.II.1).

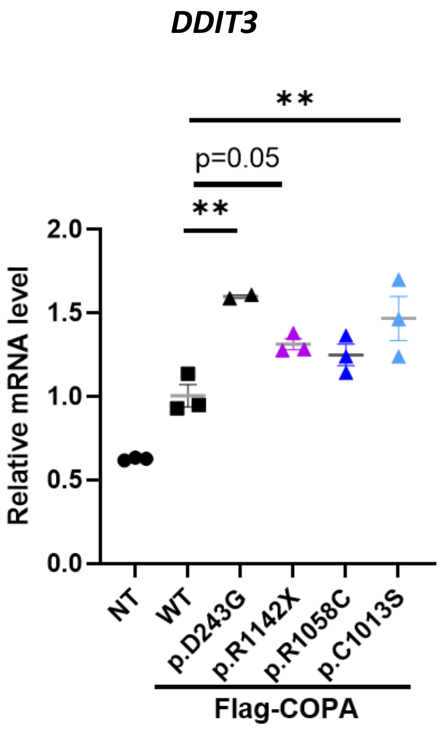

Supplemental Figure 8. **DDIT3 mRNA expression in HEK2937T cells expressing COPA mutant**. qPCR analysis of *DDIT3* mRNA expression in HEK293T cells transfected with WT COPA or mutant COPA. Results were normalized to *GAPDH* (ΔCT) and to the mean ΔCT of the control samples (ΔΔCT). Three technical replicates are shown for all except for p.D243G (n=2). Results are shown as means +/- SEM and the significance levels were calculated using one-way ANOVA test and Tukey's multiple comparisons test (*, P<0,05; **, P<0,01; ***, P<0,001; ****, P<0,0001; not significant, not depicted).

Supplemental Methods

Whole exome sequencing and sanger sequencing.

Whole exome sequencing (WES) was performed for members of family A (A.I.1, A.I.2, A.II.3 and A.II.4), family B (B.I.2 and B.II.1) and the siblings of family C (C.II.1-3), except for C.II.4. QIAamp DNA Blood Midi Kit (QIAGEN, Hilden, Germany) was used to prepare genomic DNA samples from heparinized peripheral blood. *Primary data analysis for family A.* WES libraries were prepared using SureSelect Human V4/V5 enrichment kit (Agilent Technologies, USA). One hundred fifty base pairs paired-end sequencing was performed on the Illumina NovaSeq (NIMGenetics, Madrid, Spain). BWA software was used to align the sequence reads to the Human Reference Genome Build hg19 (1). GATK Unified Genotyper was used to identify single nucleotide variants and insertions/ deletions (2). ANNOVAR was used for annotation. *Primary data analysis for family B.* The sequence reads were aligned using BWAMEM v0.7.1 to the GRCh38 reference genome (1). Duplicate reads were marked using Picard v.2.26.11 and quality control and variant joint calling of the family trios was done through GATK v4.2.5.0 (2). The variants were annotated using Variant Effect Predictor (VEP) release 105 (3). In-house scripts were used to filter variants. As previously described, while keeping the genetic hypothesis in mind, variant-level and gene-level approaches were applied to identify potential disease-causing variants (4). *Primary data analysis for family C.* WES for the paired-end pre-capture library procedure, genome DNA was fragmented by sonication and ligated to Illumina multipilexing PE adapters. The adapter ligated DNA was PCR amplified. The library was enriched by hybridizing to biotin labeled VCRome 2.1 in-solution exome probes. The post capture library DNA was subjected to sequence analysis on Illumina HiSeq platform for 100 bp paired-end reads. DNA was also analyzed by SNP-array (Illumina HumanExome-12v1 array and the SNP data compared with WES data to ensure correct identification and sequencing quality. Data analysis and interpretation was performed by Mercury. WES analysis of each patient revealed a total of 1490

variants for A.II.3, 2842 variants for B.II.1, 14 for C.II.1, 2 for C.II.2 and 11 for C.II.3.

Sanger sequencing was performed to confirm the identified COPA variants. For the members of family A (A.I.1-2, A.II.1-4) and C (C.I.1, C.I.2, C.II.1-4) genomic DNA was isolated from peripheral blood using QIAamp DNA Blood Midi Kit (QIAGEN, Hilden, Germany). For the members of family B (B.I.2 and B.II.1) complementary DNA was created after RNA isolation as described below. Primers (ThermoFisher Scientific, Waltham, USA) were designed with universal M13 tags and were constructed with the help of Primer3 (5). Primer sequences are described in supplemental material **(Supplemental Table 6).** Sanger sequencing was performed on an ABI 3730 XL Genetic Analyzer (Applied Biosystems, Waltham, USA) at the LGC Genomics Facility in Berlin, Germany. Chromas 2.6.6 was used to analyze the sequencing data.

Patients' cells, (primary) immortalized cell lines and cell culture.

HEK293T cells (ATCC) were grown in Dulbecco's Modified Eagle's Medium (DMEM, GIBCO), supplemented with 8% Fetal Bovine and Serum (FBS, Biowest), penicillin-streptomycin (100 U/ml, Life Technologies). Fibroblasts were established from skin biopsies of individual A.I.1 and patient A.II.3 and were grown in DMEM/F-12 (1:1, GIBCO) containing L-glutamine and Hepes supplemented with 10% fetal calf serum, normocin (100µg/ml, Invivogen), penicillin-streptomycin (100 U/ml, Life Technologies). EBV LCLs were created from whole blood of individual A.I.1, patient A.II.3, individual C.I.1 and patient C.II.1-4 and were grown in RPMI 1640 medium (Gibco) supplemented with 10% FBS. Creation of EBV LCLs from PBMCs of patient B.II.1 failed. All cell lines were cultured at 37°C in 5% CO2.

RNA extraction and qRT-PCR.

cDNA was created after isolation of RNA from PAXgene blood RNA tubes (BD Biosciences, New Jersey, USA) using PAXgene Blood RNA kit (Qiagen, Hilden,

Germany) for individuals of family A and C. PAX tubes were unavailable for carrier 2 (B.I.2) and patient 2 (B.II.1) and cDNA was created from their PBMCs after isolation of RNA using the PureLink RNA Mini Kit (Invitrogen, Thermo Fisher Scientific). Isolation of RNA from immortalized cell lines, HEK293T cells, fibroblasts and EBV LCLs, was performed using the latter method. mRNA was reverse transcribed with Superscript Vilo cDNA synthesis kit (Invitrogen, Thermo Fisher Scientific). qPCR was performed using SYBR green (Qiagen). The CT values obtained for the genes of interest were corrected for the cDNA input by normalization to the CT value of GAPDH (ΔCT). Furthermore, the ΔCT value was normalized to the (mean) of the control sample(s) or the EV sample (ΔΔCT). Finally, by using the formula $2^{-\Delta\Delta CT}$, the relative quantification of target cDNA is described as fold increase above control and normalized to GAPDH. A list of the used probes is supplied in supplemental material (**Supplemental Table 6**).

Western blotting.

Protein extraction for whole cell lysate analysis in PBMCs, EBV LCLs, fibroblasts and transfected HEK293T cells was performed with RIPA buffer, supplemented by 1% protease inhibitor (100X, Thermo Fisher Scientific), and 1% phosphatase inhibitor (100X, Thermo Fisher Scientific), if protein phosphorylation status was investigated. Whole cell lysates were subsequently diluted in Bolt LDS Sample Buffer (4×, Novex Life Technologies) and Bolt Sample Reducing agent (10×, Novex Life Technologies). 50µg protein lysate was resolved on 4–12% Bis-Tris Plus gels (Invitrogen). Proteins were subsequently transferred to a polyvinylidene difluoride (PVDF) membrane (Invitrogen, Fisher Scientific). Membranes were blocked for 1 hour with 5% BSA in TBS and afterwards incubated overnight with primary antibodies diluted in the blocking buffer. For evaluation of p-IRF3, membranes were blocked using LI-COR buffer for 2 hours and afterwards incubated for 48 hours in primary p-IRF3 antibody diluted in the blocking solution (6). Immunoblotting was performed with the antibodies listed in the supplemental material (**Supplemental Table 7**). Membranes were washed and probed with the

appropriate secondary HRP-coupled anti-mouse or anti-rabbit antibodies, diluted in 5% BSA in TBS, for 60 min at room temperature (RT). Bands were detected by using ECL Western blotting substrate, SuperSignal West Pico or Femto (Pierce) chemiluminescence. Signal was detected using the ChemiDOC XRS+. Western blot images were quantified with Image Lab software (Imagelab 6.1).

Plasmids.

For overexpression in HEK293T cells, WT cDNA for COPA in an untagged pCMV6-Entry backbone was purchased from Origene (SC316290). The COPA ORF encoded by the pCMV6-Entry vector was subsequently subcloned to a pCMV6-AN-DDK backbone (Origene, PS100014). The COPA cDNA was digested, with Cutsmart BufferTM (New England Biolabs (NEB)) and restriction enzymes AsiSI and MIuI-HF (NEB), from the pCMV6-Entry vector, gel-purified and ligated into the AN-DDK backbone using T4 DNA ligase (NEB), according to supplier’s instructions. Patient-specific C-terminal domain COPA mutations (p.C1013S, p.R1058C, p.R1142X) and the previously described WD40 domain COPA mutation (p.D243G) were generated by site-directed mutagenesis PCR with the CloneAmp HiFi PCR (TaKaRa). COPB2 (Origine, SC117165), COPE (Origene, SC319189) and STING (Addgene, #102598) and their corresponding EV, respectively pCMV6-XL5, pCMV6-AC and pMSCV, plasmids were purchased. The entire cDNA sequence of WT and mutant COPA, COPB2, COPE and STING were verified by Sanger sequencing in the plasmids.

Structural modelling.

The structure of the entire COPI complex is a hybrid X-ray/homology model **(Figure 2D).** The cryo-EM structure (PDB:5a1v) was used as a template for the homology modelling, sequence COPA (UniProtKB/Swiss-Prot: P53621.2), sequence COPB (UniProtKB/Swiss-Prot: P53618.3), sequence COPB’ (UniProtKB/Swiss-Prot: P35606.2) (7). The crystal structure for the C-terminal domain of COPA and COPE (pdb: 6u3v) was inserted into this model (8). The

homology modelling and virtual mutagenesis were performed with MOE (Molecular Operating Environment (MOE), 2019.01; Chemical Computing Group ULC, 1010 Sherbooke St. West, Suite #910, Montreal, QC, Canada, H3A 2R7, 2021). The figures were produced with PyMOL (The PyMOL Molecular Graphics System, Version 2.0 Schrödinger, LLC).

Intracellular fluorescent labelling, confocal microscopy.

EBV LCLs and fibroblasts were fixed and permeabilized, as described for the trafficking assays, and then incubated for 45 min with the following primary antibodies based on the type of experiment: anti-STING-TMEM173 , anti-COPI evaluated with an anti-coatomer mAb CM1A10, anti-Giantin antibody, anti-TGN46 antibody, anti-GRP78 BiP, anti-p65-NF-kB and F-actin labeled with phalloidin AlexaFluor488 **(Supplemental Table 8)**. Cells were then washed twice with 1.5 mL of saponin buffer to remove non-specifically bound antibodies and incubated with secondary antibodies for 30 minutes in a dark. Cells were washed twice in saponin buffer once with 500 µL of PBS. The labeling of the nuclei was next performed with 1 µg/mL of Hoechst for 5 min in the dark. Cells were finally washed twice with 1.5 mL PBS, deposited on slides and mounted with FluorSaveTM Reagent (Millipore).

For immunofluorescence of HEK293T, cells were plated at ~75% confluency in 12-well dishes containing a gelatin coated glass coverslip. Cells were transfected using Fugene HD (Promega). 48 hours post transfection, cells were fixed in well with 4% paraformaldehyde at RT to 20 min then permeabilized with Triton 0.1% at RT for 30 min. Blocking was performed with 3% BSA-PBS-T for 30 min. Primary antibody was diluted in 3% BSA-PBS-T and incubated on cells for 1.5 hour at RT, cells were washed 3 times with PBS-T, then incubated with secondary antibody diluted in 3% BSA-PBS-T for 45 minutes. Cells were then washed 3 times with PBS-T and cover slips were mounted in VECTASHIELD Hardset Antifade (Vector

Labs) for imaging. Primary antibodies used included anti-STING D2P2F, anti-FLAG M2, Calnexin AF18 and GM130.

Microscopy image analysis

Images were obtained with an inverted fluorescence microscope (Eclipse Nikon TE300), a Photometrics Cascade camera, and acquired using Metamorph v7.8.9.0 software. A 100x objective was used for EBV LCL cells and a 40X objective for fibroblasts. Image analyses were performed using Fiji software (ImageJ version 1.51u). Pearson coefficient (PC) was measured in Fiji by using a macro containing the Coloco2 plugin. This coefficient measures the degree of overlap between the two stains and the Golgi apparatus or endoplasmic reticulum. A PC value of 0 means that there is no colocalization between the 2 stains. By contrast, a PC value of 1 means that there is a perfect colocalization between the two stains taken into account. The analysis of the integrated intensity in HEK293T cells was determined using ImageJ v1.51 (NIH) of TIFF files. 2-4 cells were used from each TIFF from 3 separate transfections. A 20x20 pixel area was measured over an area of STING-488 expression, with an area corresponding to the inset image in the figure taken to match the representative image. Images were taken 400X on a Nickon Ti Elipse. Image comparison was performed from images taken with the same exposure and linear adjustment. Fluorescence intensity was calculated using corrected total fluorescence of a 100 × 100-pixel area using: integrated density − [(selected area) × (mean fluorescence of background readings)].

p65 nuclear translocation in fibroblasts.

To evaluate the nuclear translocation of p65 by immunoblotting, fibroblasts of patient A.II.3, carrier A.I.1 and a healthy control were seeded in a 6-well plate containing 400 000 cells/well in DMEM/F-12 medium containing 2.5% FCS. 24 hours after seeding, cells were stimulated with TNF-α (100ng/ml, Invivogen) for 120, 60, 30 minutes or remained unstimulated. Cytoplasmic and nuclear cell extract were extracted using the reagents and protocol of the NE-PER Nuclear and

Cytoplasmic Extraction kit (Thermo Fisher Scientific). Western blot was performed as described above. To evaluate nuclear translocation of NF-κB in fibroblasts by confocal microscopy, cells were stimulated with LPS (2µg/mL, Sigma, L6529) for one hour. Intracellular fluorescent staining and microscopy image analysis were performed as described above.

Electron microscopy.

Fibroblasts of a healthy control and patient A.II.3 were pelleted and fixed in 4% paraformaldehyde and 2.5% glutaraldehyde in 0.1M Sodium Cacodylate buffer, and after washing in the same buffer, subsequently treated with 1% osmim tetroxide and 1.5 mM ferrocyanide in 0.1M sodium cacodylate buffer. Then, after washing in 0.1M cacodylate buffer, the samples were stained with 1% uranyl acetate and lead aspartate. Finally, the samples were rinsed and dehydrated in a graded ethanol series from 30-100% after which they were washed in propylene oxide and embedded in Epoxy resin. Ultrathin sections (70 nm) were cut with a Leica ultracut S ultramicrotome and some of them post-stained in uranyl acetate and Reynold's lead citrate. The sections were examined and images taken with a JEOL JEM1400 (Tokyo, Japan) transmission electron microscope equipped with an 11 Mpxl EMSIS Quemesa camera.

Cytokine evaluation in serum samples

The MSD V-plex Proinflammatory Panel 1 Human kit (MSD platform, Rockville, Maryland, USA) was used to measure serum IFN-γ, IL-1β, IL-2, IL-4, IL-6, IL-8, IL-10, IL-12p70, IL-13 and TFN-α concentrations (pg/ml) according to the manufacturer's instructions. The lower limits of detection (LLOD) for the cytokines ranged between 0.01–0.89 pg/ml.

Flowcytometry

Few PBMCs of patient A.II.3 were available to perform flowcytometry. Unfortunately, PBMCs were sparse for the other patients. We evaluated pSTAT1 in PBMCs by flowcytometry as previously described (9–11). Mononuclear cells were

suspended at 1.10^6 cells/ml in serum-free RPMI medium. Cells were stimulated with IFN-γ (1000 U/ml) for 15 minutes. A single condition was pretreated with pervanadate (100X), a phosphatase inhibitor (12). After 15 minutes, they were washed and stained for CD14 APC-eF780 antibody (Invitrogen, Thermo Fisher Scientific). 3 conditions were simultaneously incubated with staurosporine (0.5µM, BD biosciences) for 45 min, 30 min or 15 min in RPMI. Afterwards cells were fixed and permeabilized with 80% ice cold methanol. Finally they were stained with PE-conjugated anti-pSTAT1 (BD Biosciences). Furthermore, the total amount of monocytes, the monocyte subpopulations and the expression of CD169/SIGLEC-1 was analyzed in PBMCs of patient A.II.3 and 8 healthy donors, as previously described (13). Routine immunophenotyping and T helper cell staining was performed as previously described (13). The used antibodies are listed in **Supplemental Table 7.** Flow Cytometry was performed on the Canto II and subjected to flow cytometric analysis using Flowjo Software.

Bulk RNA sequencing

RNA was isolated from PAX tubes, as described above, from 2 healthy control children (1 male and 1 female), 2 adult healthy control (1 male and 1 female), carrier A.I.1, patient A.II.3, carrier C.I.1, patients C.II.1-4 and one SAVI patient, heterozygous for the c.463G>A (p.V155M) variant in STING, as a control. The quality and the yield of the RNA was assessed using NanoDrop 8000 Spectrophotometer (Thermo Scientific). 100 to 250ng of RNA was used as input for the Lexogen QuantSeq 3' mRNA libraries. These libraries were sequenced on a Illumina HiSeq4000 generating single-end 51bp reads. Optical duplicates and adaptors were removed with clumpify and fastx-toolkit, respectively. Next the reads were mapped to the human reference genome with Star 2.6. and gene expression matrices were generated with HTSeq (version 0.10.0) (14, 15). Data were further analyzed using R and DESeq2 (16). Principal component analysis was performed to select appropriate groups to perform differential gene expression analysis. Heatmaps were generated using R and by creating a normalized count matrix (via

DESeq2), performing a variance stabilizing transformation, and finally scaling each gene so the mean and variance are zero and one respectively. IPA software (Qiagen) was used to perform the differential gene expression analysis and for data visualization. A cutoff on the adjusted p-value of 0.05 has been selected (or false discovery rate of 5%) for the analysis. IPA analysis of the differential gene expression analysis for patient A.II.3 and patient C.II.4 versus the group consisting of the carriers (A.I.1 and C.I.1), controls (n=4), SAVI patient and patient C.II.1-3 withheld respectively 698 and 2923 genes, who passed the cut-off of the adjusted p-value of 0.05.

Case reports

Patient 1 (A.II.3), COPA^{R1142X}

The patient is the third child of a non-consanguineous couple of Caucasian descent. She was born term following a normal pregnancy. At the age of 8 years old she suffered from a first episode of optic neuritis. The presence of aquaporine-4 antibodies in the serum prompted the diagnosis of Neuromyelitis Optica Spectrum Disorder and treatment with Mycophenolic acid was started. Despite this treatment she experienced a relapse of chiasmic neuritis at the age of 9 years old. 6-monthly intravenous administration of Rituximab was associated to the treatment regimen. Treatment with Mycophenolic acid was interrupted due to side effects. At the age of 11 years old a new episode of transverse myelitis required again the association of Mycophenolic acid to the treatment with Rituximab. Hepatosplenomegaly, associated with a progressive increase of the liver tests, and intra-abdominal lymphadenopathy was detected at the age of 12 years old and evolved into cirrhosis with portal hypertension and esophageal varices. Progressive pancytopenia developed and bone marrow dysplasia was noted. Liver and spleen biopsy did not reveal signs of Hemophagocytic Lymphohistiocytosis (HLH). Additional treatment with Rapamycin and Ruxolitinib did not result in disease control. Disease activity responded only to high dose corticosteroid treatment with relapse upon tapering. At

the age of 15 years old she was hospitalized at the intensive care unit (ICU) due to an alveolar hemorrhage. She was treated with a hematopoietic stem cell transplantation at the age of 15 years and 6 months due to insufficient disease control despite extensive immunomodulatory treatment. She is now 14 months out of HSCT, with 81% donor chimerism yet still incomplete immune reconstitution (data not shown).

Patient 2 (B.II.1), COPA^{R1058C}

This patient is the first child of a non-consanguineous couple of African American descent. The patient presented with livedo reticularis, acrocyanosis and heat sensitivity at age 5. He also developed increased somnolence and unusual sleep-wake cycles. At the age of 6 years and 2 months he was admitted to the ICU due to respiratory failure and autonomic instability, including hypothermia, bradycardia and hypotension. He was diagnosed with an alveolar hemorrhage and found to have positive Anti-neutrophil cytoplasmic antibodies (ANCA) and anti-OJ IgG, although he had normal strength and muscle enzymes were normal. Since then, he suffered from recurrent alveolar hemorrhage with need of ICU hospitalization and mechanical ventilation. His condition worsened despite treatment with corticosteroids, Rituximab, Azathioprine, Cyclophosphamide, Anakinra, and Ruxolitinib. One and a half years after his initial presentation he succumbed due to multi-organ failure, Candida parapsilosis fungemia and macrophage activation syndrome, despite treatment with pulse steroids and Emapalumab.

Patient 3-6 (C.II.1-4), COPA^{C1013S}

Patient C.II.1, patient 3, presented at the age of 5 years old with IgA nephropathy and hypogammaglobulinemia. He was successfully treated with corticosteroids and Azathioprine. He experienced recurrences of proteinuria which necessitated treatment with corticosteroids, Rituximab and Azathioprine. His kidney biopsies did not reveal active inflammation, vasculitis, nor recurrence of crescentic disease. Despite significant improvement of the proteinuria, the hypogammaglobulinemia persisted and was treated with intravenous immunoglobulin (IVIG). At the age of

13 years old he experienced bilateral knee arthralgia without joint swelling. Auto-antibodies were absent, while his antinuclear antibody (ANA) titer was weakly positive (1:160) and showed a speckled, nucleolar pattern. Chest CT scan, performed as a part of the diagnostic work-up of the discovered COPA variant, revealed few scattered subcentimeter pulmonary nodules. Patient C.II.2, patient 4, the younger sister of patient 3, was diagnosed at the age of 12 years old with a neuroblastoma. During her follow-up she suffered from diffuse joint pain and chronic abdominal pain. Intestinal biopsies revealed chronic inflammation with infiltrating eosinophils. At the age of 17 years old she developed hypertension and stage II kidney disease, complex regional pain syndrome and vasculitis of the legs. Patient 5, C.II.3, and 6, C.II.4, are prematurely born, dizygotic, twins and experienced respiratory tract infections such as recurrent acute otitis media and pneumonia. At the age of 2 years old, patient 5, C.II.3, presented with insidious onset left hand weakness and chronic thoraco-lumbar back pain at night. His symptoms progressed such that he had urinary incontinence, night-time awakenings due to pain, and intermittent fevers. MRI spine and brain were not suggestive of neuroblastoma. He experienced episodes of back pain, often triggered by acute respiratory illnesses, with resolution after treatment with non-steroidal anti-inflammatory drugs. His autoantibody workup was negative. His chest CT scan was unremarkable. The neonatal period of patient 6, C.II.4, was complicated by an alveolar hemorrhage. Further, patient 6 experienced recurrent episodes of arthralgia of the hip and knee with resolution of the symptoms after treatment with non-steroidal anti-inflammatory drugs.

References

1. Li H, Durbin R. Fast and accurate long-read alignment with Burrows-Wheeler transform. *Bioinformatics* 2010;26(5):589–595.

2. McKenna A et al. The Genome Analysis Toolkit: A MapReduce framework for analyzing next-generation DNA sequencing data Aaron. *Genome Res* 2009;254–260.

3. McLaren W et al. The Ensembl Variant Effect Predictor. *Genome Biol* 2016;17(1):1–14.

4. Bucciol G, Van Nieuwenhove E, Moens L, Itan Y, Meyts I. Whole exome sequencing in inborn errors of immunity: Use the power but mind the limits. *Curr Opin Allergy Clin Immunol* 2017;17(6):421–430.

5. Kõressaar T et al. Primer3-masker: Integrating masking of template sequence with primer design software. *Bioinformatics* 2018;34(11):1937–1938.

6. Lepelley A et al. Mutations in COPA lead to abnormal trafficking of STING to the Golgi and interferon signalling. *J. Exp. Med.* 2020;217(11).

7. Dodonova SO et al. A structure of the COPI coat and the role of coat proteins in membrane vesicle assembly. *Science (1979)* 2015;349(6244):195–198.

8. Travis SM, Kokona B, Fairman R, Hughson FM. Roles of singleton tryptophan motifs in COPI coat stability and vesicle tethering. *Proc Natl Acad Sci U S A* 2019;116(48):24031–24040.

9. Mizoguchi Y et al. Simple diagnosis of STAT1 gain-of-function alleles in patients with chronic mucocutaneous candidiasis. *J Leukoc Biol* 2014;95(4):667–676.

10. Soltész B et al. New and recurrent gain-of-function STAT1 mutations in patients with chronic mucocutaneous candidiasis from Eastern and Central Europe. *J Med Genet* 2013;50(9):567–578.

11. Liu L et al. Gain-of-function human STAT1 mutations impair IL-17 immunity and underlie chronic mucocutaneous candidiasis. *Journal of Experimental Medicine* 2011;208(18):1635–1648.

12. O'Shea JJ, Mcvicar DW, Bailey TL, Burns C, Smyth MJ. Activation of human peripheral blood T lymphocytes by pharmacological induction of protein-tyrosine phosphorylation. *Proc Natl Acad Sci U S A* 1992;89(21):10306–10310.

13. Yap JY et al. Intrinsic Defects in B Cell Development and Differentiation, T Cell Exhaustion and Altered Unconventional T Cell Generation Characterize Human Adenosine Deaminase Type 2 Deficiency. *J Clin Immunol* 2021;41(8):1915–1935.

14. Dobin A et al. STAR: Ultrafast universal RNA-seq aligner. *Bioinformatics* 2013;29(1):15–21.

15. Anders S, Pyl PT, Huber W. HTSeq-A Python framework to work with high-throughput sequencing data. *Bioinformatics* 2015;31(2):166–169.

16. Love MI, Huber W, Anders S. Moderated estimation of fold change and dispersion for RNA-seq data with DESeq2. *Genome Biol* 2014;15(12):1–21.
